# Prognostic performance of bedside tests for predicting ulcer healing and wound healing after minor amputation in patients prone to medial arterial calcification: A systematic review

**DOI:** 10.1177/1358863X241309326

**Published:** 2025-01-21

**Authors:** Siem A Willems, Jelle A Nieuwstraten, Abbey Schepers, Jan van Schaik, Pim van den Hoven, Joost R van der Vorst, Jaap F Hamming, Jeroen JWM Brouwers

**Affiliations:** Department of Vascular Surgery, Leiden University Medical Center, Leiden, The Netherlands

**Keywords:** diabetes mellitus, medial arterial calcification, peripheral artery disease (PAD), systematic review, ulcer healing

## Abstract

Foot ulceration is a significant and growing health problem worldwide, particularly due to rises in diabetes mellitus (DM) and peripheral artery disease. The prediction of ulcer healing remains a major challenge. In patients with foot ulcers, medial arterial calcification (MAC) can be present as a result of concomitant DM or chronic kidney disease and is a prognostic factor for unfavorable outcome. This systematic review aimed to evaluate the prognostic reliability of bedside tests to predict ulcer healing and wound healing after minor amputation in patients prone to MAC, following PRISMA guidelines. Primary endpoints were the positive and negative likelihood ratios for ulcer healing. Methodological quality and risk of bias were assessed using the QUIPS-tool. A total of 35 studies were included, predominantly investigating transcutaneous oxygen pressure (TcPO_2_), followed by ankle–brachial index and toe pressure. None of these bedside tests effectively provided an acceptable trade-off between predicting healing and nonhealing. A TcPO_2_ below 30 mmHg was most closely associated with nonhealing of an ulcer. The same applied to wound healing after minor amputation, in which none of the bedside tests was able to sufficiently predict healing or nonhealing. To conclude, currently used bedside tests lack acceptable prognostic performance for ulcer healing and healing after minor amputation in patients prone to MAC. Future prospective studies should establish a clear definition of ulcer healing, utilize a standardized wound classification system, and minimize patient heterogeneity. A combined assessment of microvascular and macrovascular perfusion status could improve the prediction of wound healing.

## Background

Patients with foot ulceration are a growing medical concern worldwide, particularly due to an increase in diabetes mellitus (DM), chronic limb-threatening ischemia (CLTI), or a combination of both.^1,2^ In addition, peripheral artery disease (PAD) is present in up to 50% of patients with diabetic foot ulcers (DFU).^3^ Owing to demographic trends towards aging and projected rises in important risk factors for PAD, these patient groups are rapidly expanding.^4^ Previous studies showed that the overall rate of ulceration recurrence, risk of limb loss, and mortality are strikingly high, causing serious impact on the quality of life in these patients.^5,6^ Therefore, urgent referral to a vascular specialist is of great importance to perform hemodynamic testing, accurately stage the severity of ulceration, and to assess the need for revascularization.

In patients with DM and/or PAD, medial arterial calcification (MAC) can be present. MAC is a systemic vascular disorder in which calcification of the tunica media results in arterial stiffness.^7^ This process is believed to be associated with aging, but is expedited in patients with DM and chronic kidney disease (CKD).^8–10^ Patients with MAC have a notably higher risk of cardiovascular mortality and limb events.^11–13^ Previous research also indicates that MAC is strongly associated with a higher risk of major lower-limb amputation in patients with PAD.^14^ Hence, it is of utmost importance to adequately stage PAD in order to manage foot ulcers in patients with MAC. However, it is well-known that the diagnostic performance of bedside tests to detect PAD can be diminished in these patients.^15–17^ An underestimation of its severity could potentially lead to incorrect assumptions about wound ischemia as well.

Currently, a variety of bedside tests are included in the work-up of patients referred for suspected PAD.^18^ These include, among others, ankle–brachial index (ABI), toe–brachial index (TBI), toe pressure (TP), and transcutaneous oxygen pressure (TcPO_2_). These bedside tests are not only used to diagnose PAD but are also used to predict wound healing. However, in patients prone to MAC (e.g., DM or CKD), it is unclear if these tests are sufficiently accurate to also predict (non)healing of foot ulcers or minor amputations wounds. Therefore, we conducted a systematic review evaluating the prognostic performance of various bedside tests for the prediction of ulcer healing in patients prone to MAC. In addition, wound healing after minor amputation due to a previously diagnosed foot ulcer was also investigated.

## Methods

### Search strategy

**Figure 1.**
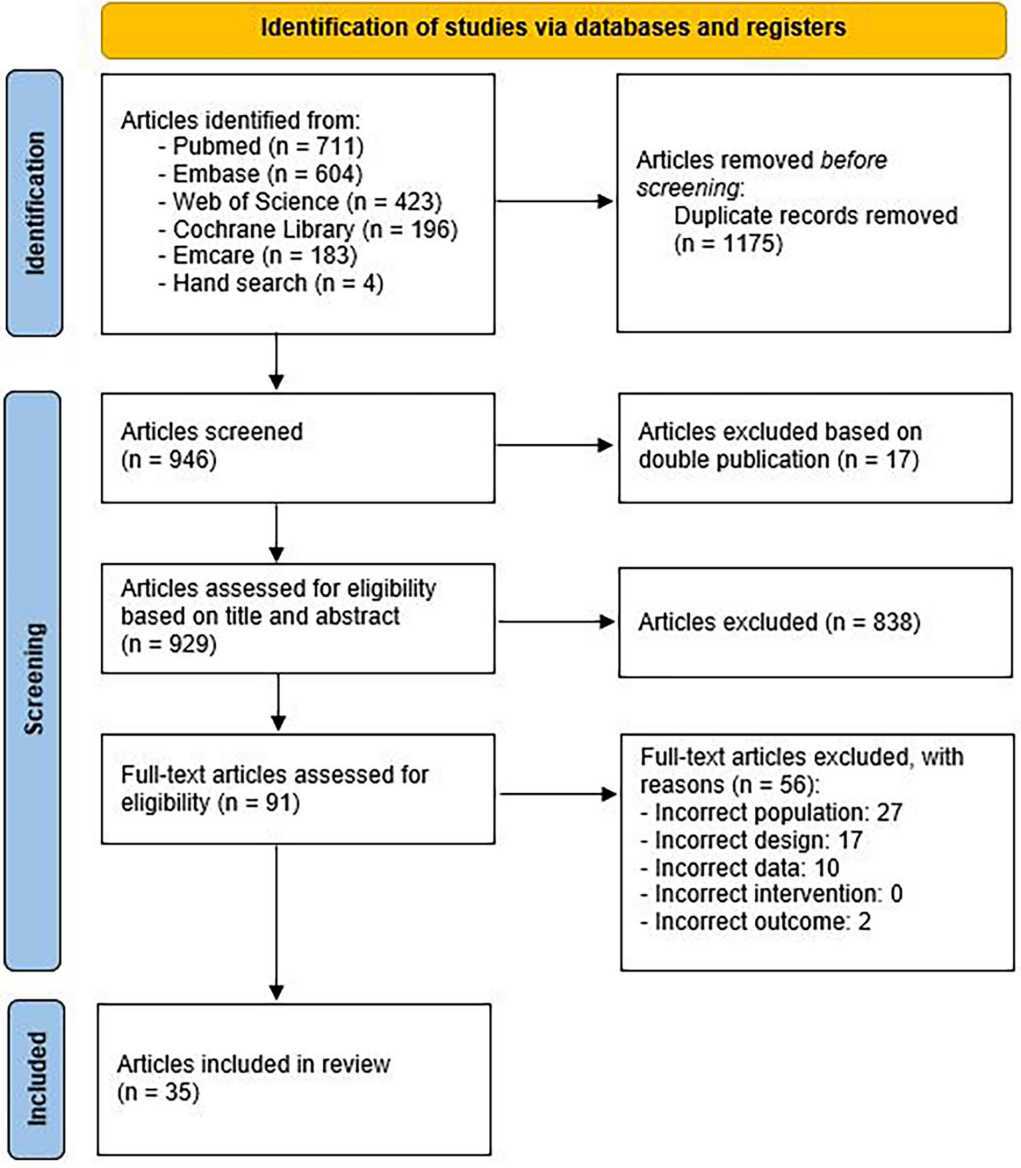
Flow diagram illustrating the inclusion process according to PRISMA guidelines.

This study was conducted according to the PRISMA (Preferred Reporting Items for Systematic reviews and Meta-Analyses) guidelines.^19^ A literature search was performed in PubMed, Embase (OVID-version), Web of Science, Cochrane Library, and Emcare for articles published before June 2023. A set of 10 key publications was used to validate the search string. Two reviewers (SW, JN) independently screened the titles and abstracts for eligibility of inclusion. Dissimilarities were resolved in a discussion meeting between two reviewers (SW, JN) and, if needed, deliberated with a third author (JB). The selected abstracts were independently assessed for definitive inclusion based on full text reading, and the data were extracted. A flow diagram (Figure 1) was maintained for transparency. The search strategy can be found in Supplement S1.

### Selection criteria

We aimed to evaluate the prognostic performance of bedside tests to predict (nontraumatic) ulcer healing in patients prone to MAC. Studies investigating primary ulcer healing or wound healing after minor amputation (up to transmetatarsal) were included. Bedside tests were identified as any noninvasive tool that is easily available at the point-of-care. To be eligible for inclusion, studies needed to match the following criteria: (i) evaluated a bedside test (e.g., ABI, TBI, ankle pressure (AP), TP, TcPO_2_) and (ii) included patients (in the subanalyses) who were prone to MAC, defined as DM, CKD, or ABI > 1.3. If revascularization procedures were performed during the study, new measurements of the bedside test had to be performed after revascularization in order to be included. We excluded articles that reported insufficient data or were case reports. Studies that investigated healing of major amputations were also excluded as the bedside tests are not measured at the level of these amputations.

### Data extraction and statistical analysis

Data extraction was performed by two investigators (SW, JN) independently. If an article was eligible for inclusion, the extracted data contained relevant patient characteristics, the bedside test investigated, and the prognostic performance of that test. In case these parameters were not specified, but could be calculated from the accessible data, data extraction was undertaken. The primary outcomes of interest regarding prognostic performance of ulcer healing were the positive likelihood ratio (PLR) and negative likelihood ratio (NLR). In this review, the PLR reflected the chance of ulcer healing, whereas the NLR indicated that the ulcer did not heal during follow-up. The interpretation of these likelihood ratios is shown in Table 1.^20^ We classified the trade-off between PLR and NLR as poor (PLR < 5 and NLR > 0.2), moderate (PLR 5–10 and NLR 0.1–0.2), or excellent (PLR > 10 and NLR < 0.1). Sensitivity and specificity were included in the final evidence table as well. If articles presented their prognostic performance as the chance of nonhealing instead of healing (of an ulcer or minor amputation wound), a recalculation was performed to equally compare the results.

**Table 1.** Interpretation of likelihood ratios and their effect on the posttest probability of disease.

Positive likelihood ratio (PLR)	Negative likelihood ratio (NLR)	Interpretation: effect on ability to rule in/rule out disease
> 10	< 0.1	Large
5–10	0.1–0.2	Moderate
2–5	0.2–0.5	Small
1	1	No change

### Quality assessment

Methodological quality and risk of bias was assessed using the Quality in Prognosis Studies (QUIPS) tool.^21^ This tool incorporates six domains for critical assessment of study validity and bias including study participation, study attrition, prognostic factor measurement, outcome measurement, study confounding, and statistical analysis. All domains were judged as low, moderate, or high risk of bias. For inclusion in this review, no minimal level of methodological quality was needed. Owing to heterogeneity in patient selection, study design, and different primary outcome measures, a meta-analysis could not be performed.

## Results

### Search results

In total, 2121 articles were identified. After removal of duplicates and double publications, 929 studies were assessed for eligibility based on title and abstract. Of these, 91 articles were considered appropriate for full-text review, and 35 could eventually be included in this review. In total, 27 articles evaluated primary ulcer healing and eight studies investigated wound healing after minor amputation. The PRISMA flow diagram of this process is illustrated in Figure 1.

### Overview of studies

A total of 6298 patients were incorporated in this review. The median age of patients was 65 years old (range 57-77 years old) and the majority of patients were men. Five articles did not provide any information about the demographic data of their patient population.^22–26^ Except for one article regarding primary ulcer healing in patients with chronic renal failure,^23^ all studies evaluated DFU or minor amputation wound healing in patients with DM. Classification of these ulcers was presented quite heterogeneously, whereas most studies used the Wagner Scale or University of Texas Staging System. Overall, primary healing of ulcers varied greatly (between 38% and 83%) with different follow-up periods across the studies. In total, almost half of the articles (16) were published before 2000. Study characteristics and extensive details of the 35 included studies can be found in Tables S2 and S3. Methodological assessment according to the QUIPS-tool is provided in Table 2.

**Table 2.** Methodological assessment of all included studies based on the QUIPS-tool (*N* = 35 studies).

Reference	Participation	Attrition	Prognostic factor measurement	Outcome measurement	Study confounding	Statistical analysis and reporting
Apelqvist 1989^27^	+++	+++	+++	+++	++	++
Apelqvist 1990^28^	+++	+++	+++	+++	+	+
Ballard 1995^29^	++	+++	+++	+++	++	+++
Bishara 2009^30^	+++	+++	+++	+++	+++	+++
Brechow 2013^37^	+++	+	++	+	+	+
Elghazaly 2023^31^	+++	+++	+++	+++	+++	+++
Elgzyri 2013^47^	++	++	++	++	+	++
Elgzyri 2021^38^	+++	++	++	++	+++	+++
Faris 1985^39^	+	++	+	+	+	+
Gibbons 1979^50^	+	+	++	++	+	+
Holstein 1980^40^	+	+	++	+	+	++
Holstein 1984^51^	++	++	+	+	++	+
Kalani 1999^44^	+	++	++	+	+	++
Karanfilian 1986^45^	+	+	++	+	+	++
Kawai 2017^22^	+	++	+++	+	+	++
Ladurner 2010^32^	+	+	++	++	++	++
Larsson 1993^52^	++	++	++	++	+	++
Lee 2019^48^	+++	++	+++	+++	++	+++
Lee 2022^49^	+++	++	+++	+++	++	+++
Leenstra 2020^33^	++	+++	+++	+++	+++	+++
López-Moral 2022^34^	+	+++	+	+++	++	+++
Manu 2021^41^	++	++	+++	++	++	+++
Mehta 1980^53^	++	++	++	+	+	+
Mennes 2021^35^	++	++	++	+++	++	+++
Nouvong 2009^36^	++	+	++	+++	+	++
Padberg 1996^23^	++	+++	++	++	+	++
Rajagopalan 2018^42^	++	++	++	++	++	++
Thottiyen 2023^46^	++	+++	++	++	++	++
Vincente Jiménez 2015^24^	+	+++	+++	+	++	+
**Vitti 1994^54^**	+++	++	+++	+	+	+
**Wallin 1989^43^**	++	+++	+++	+	++	+
**Welch 1985^55^**	++	+	+++	++	+++	+
**Wyss 1988^25^**	+	+	+	+	+	++
**Yang 2013^26^**	++	+++	+++	++	++	+++
**Zhang 2019^56^**	+++	++	+++	++	+	++

All domains were rated according to their potential risk of bias and classified as: +, high risk of bias; ++, moderate risk of bias; +++, low risk of bias.

QUIPS-tool, Quality in Prognosis Studies tool.

### Overview of prognostic bedside tests

Most of the studies looked at multiple bedside tests regarding primary ulcer healing. TcPO_2_ was investigated most frequently (13 studies), followed by ABI (10 studies), TP (eight studies), and AP (seven studies). Other bedside tests were skin perfusion pressure (three studies), palpable pulses (two studies), and skin hydration level (two studies).

When looking at healing minor amputation wounds, the most commonly studied bedside test was AP (five studies), followed by TP (three studies) and ABI (two studies). A comprehensive summary of all these tests is shown in Table 3 and the subparagraphs below. Additionally, Table 4 shows an overview of the key features of the most commonly used bedside tests regarding ulcer healing and wound healing after minor amputation.

**Table 3.** Summary of evidence for the prognostic performance of different bedside tests with corresponding cut-off values.

Bedside test	Cut-off value	Studies	Primary ulcer healing		Studies	Minor amputation	
			PLR	NLR		PLR	NLR
**ABI**	> 0.40				1^61^	1.3	0.07
	⩾ 0.50	2^28,42^	1.1, 1.2	0.90, 0.61	1^57^	2.0	0.12
	⩾ 0.52	1^39^	4.0	0			
	⩾ 0.60	1^34^	1.6	0.15			
	⩾ 0.65	1^51^	3.6	0.18			
	> 0.70				1^61^	4.6	0.2
	⩾ 0.90	6^36,40–42,46,47^	1.05, 1.9, 1.1, 1.7, 1.1, 1.53	0.92, 1.00, 0.78, 0.61, 0.48, 0.25	1^61^	Infinite	0.87
	> 1.10	1^28^	1.0	0.98			
**Ankle pressure**	⩾ 30	1^50^	0.8	2.35			
	⩾ 40	1^32^	1.1	0.00	1^60^	1.0	1.1
	⩾ 50	3^36,45,52^	0.5, 1.1, 1.3	1.08, 0.48, 0	1^56^	1.1	0^*^
	⩽ 50	1^36^	0.9	2.2			
	> 60				2^58,60^	1.2, 1.0	0.98, 0.9
	⩾ 70	1^48^	3.4	0.10	1^55^	1.0	1.09
	> 75				1^57^	2.0	0.16
	⩾ 80	3^32,43,45^	1.5, 1.0, 2.7	0.30, 1.09, 0.28			
	⩾ 96	1^40^	1.5	0.4			
**TBI**	> 0.10				1^57^	1.9	0.09
	> 0.51	1^40^	1.5	0.53			
	> 0.65	1^39^	0	0.28			
	⩾ 0.75	1^46^	0.9	1.05			
	⩾ 0.80	1^36^	2.4	0.63			
**Toe pressure**	> 15	1^32^	1.4	0.1	1^57^	1.7	0.10
	⩾ 20	1^48^	4.1	0.2			
	⩾ 30	4^43,45,49,52^	1.1, 0.7, infinite, 5.0	0.88, 1.66, 0.28, 0.88	1^56^	1.3	0.66
	> 38				1^59^	2.3	Infinite
	⩾ 40	1^36^	0.92	1.08			
	> 45	2^32,49^	3.1, 2.9	0.30, 0.64			
	> 54	1^40^	1.5	0.40			
**TcPO_2_**	> 10	1^50^	6.0	0			
	> 20	2^28,37^	1.7, 2.4	0.43, 0.58			
	> 25–30.5	7^28,31,34,39,40,49,51^	4.3, 5.0, 1.8, 0, 1.2, 6.0, 4.7	0.23, 0.14, 0.05, 0.09, 0.47, 0.09, 0.18			
	⩾ 31				1^30^	5.9	0.2
	⩾ 40	4^36,38,47,53^	1.4, 2.8, 1.5, 1.8	0.70, 0.67, 0.36, 0.39			

In this review, the PLR reflected the chance of wound healing, whereas the NLR indicated the chance that the wound would not heal during follow-up. Studies are sorted alphabetically then ranked according to the reported likelihood ratios.

* In the corresponding study, no wound healing was observed below the reported cut-off value, resulting in an NLR of 0.

ABI, ankle–brachial index; NLR, negative likelihood ratio; PLR, positive likelihood ratio; TBI, toe–brachial index; TcPO_2_, transcutaneous oxygen pressure.

**Table 4.** Overview of the key features of commonly used bedside tests regarding ulcer healing and wound healing after minor amputation.

Bedside test	Technology	Strengths	Weaknesses
**ABI**	Doppler ultrasound; index comparison between brachial and ankle blood pressure; external pressure measurement	– High ABIs (> 0.7) seem to predict healing after minor amputation	– No acceptable trade-off between PLR/NLR for different cut-off values – No clear correlation between higher/lower cut-off value and prognostic performance
**Ankle pressure**	Measurement of absolute blood pressure	– Most studied bedside tests for healing after minor amputation	– No acceptable trade-off between PLR/NLR for different cut-off values – No clear correlation between higher/lower cut-off value and prognostic performance
**TBI**	Doppler ultrasound; index comparison between brachial and toe blood pressure; external pressure measurement	– None	– Few conducted studies – Bad prognostic performances for both primary healing and healing after minor amputation
**Toe pressure**	Measurement of absolute blood pressure	– Lower toe pressures seem to correlate with lower chances of wound healing (i.e., < 30 mmHg)	– Higher toe pressures do not sufficiently predict ulcer healing – No acceptable trade-off for adequate prognostic performance
**TcPO_2_**	Heated electrode to assess oxygen levels just below the skin’s surface	– Most studied bedside tests for primary ulcer healing – Lower TcPO_2_ values seem to correlate with lower chances of wound healing (i.e., < 30 mmHg)	– Prediction for healing after minor amputation not known – Higher TcPO_2_ do sufficiently predict ulcer healing

ABI, ankle–brachial index; NLR, negative likelihood ratio; PLR, positive likelihood ratio; TBI, toe–brachial pressure; TcPO_2_, transcutaneous oxygen pressure.

### Definition of primary ulcer healing

Different definitions of primary ulcer healing were seen across the studies. Intact skin with full epithelization within 6–12 months was most frequently stated as ulcer healing (11 studies).^23,27–36^

Seven of the studies regarded healing after the need for a minor amputation as primary ulcer healing as well.^37–43^ Improvement of the wound (reduction in size, granulation tissue) was applied as the definition in four studies.^26,44–46^ Five studies did not specify the definition of ulcer healing.^24,32,47–49^

### Quality assessment of included studies

The QUIPS assessment of study validity and risk of bias is shown in Table 2. The quality of studies was generally poor. Only two articles had a low risk of bias on all six domains.^30,31^ Risk of bias was usually moderate or high with respect to study participation, outcome measurement, and study confounding. As an example, the definition of ulcer healing (outcome measurement) and respective follow-up time varied greatly, which led to differences in prognostic performances across the studies. Also, patient selection and ulcer characteristics (including presence of foot infection and neuropathy) were diverse. In general, the scientific evidence for all studies as a whole was rated as low.

### Primary ulcer healing

#### ABI and ankle pressure

A total of 10 studies investigated ABI as a predictor of ulcer healing.^29,31,34–37,41,42,45,46^ Various cut-off values were used (⩾ 0.50 twice, > 0.52 once, ⩾ 0.60 once, > 0.65 once, ⩾ 0.90 six times, and > 1.10 once). Studies from Thottiyen et al. (ABI > 0.65), López-Moral et al. (ABI > 0.52), and Ballard et al. (ABI ⩾ 60) found good NLRs of 0.18, 0.0, and 0.15 and PLRs of 3.6, 4.0, and 1.60, respectively.^29,34,46^ Despite the good NLRs, there was no acceptable trade off compared to the PLRs. Moreover, the NLR in the study of López-Moral et al. was merely 0 because there was no healing of wounds observed below ABI 0.52. Therefore, the NLR could not be calculated properly. None of the other studies with different cut-off values was able to demonstrate an acceptable trade-off in predicting ulcer healing. PLRs ranged from 1.06 to 1.9 compared to NLRs ranging from 0.25 to 1.0, indicating insufficient prognostic performance. A comprehensive overview is shown in Table 3.

Ankle pressure was assessed as a predictor for ulcer healing in seven studies.^27,31,35,38,40,43,47^ Cut-off values used were ⩾ 30 (once), ⩾ 40 (once), ⩾ 50 (thrice), ⩾ 70 (once), ⩾ 80 (thrice), and > 96 (once) mmHg. Wallin et al. reported the best results for predicting ulcer healing with a cut-off value above 70 mmHg, showing a PLR of 3.4 (NLR 0.1).^43^ However, both primary ulcer healing and wound healing after minor amputation were considered as healing, which probably led to an overestimation of the prognostic performance. None of the remaining studies with previously mentioned cut-off values provided an acceptable trade-off in predicting healing versus nonhealing. PLRs and NLRs ranged from 0.8 to 2.7 and 0 to 2.35, respectively. NLR was 0 in the study of Apelqvist et al., mainly because none of the wounds healed in patients with an AP below 40 mmHg.^27^ Elghazaly et al. performed a prospective cohort study with the highest degree of methodological quality, investigating AP with a cut-off value below 50 mmHg.^31^ This resulted in a PLR of 0.50 and a NLR of 0.92. These results indicate insufficient prognostic performance.

#### Toe–brachial index and toe pressure

In total, four studies investigated the TBI as predictor for wound healing.^31,34,35,41^ All of these studies used different cut-off values (0.51, 0.65, 0.75, and 0.8); however, none was able to establish a reasonable trade-off between predicting ulcer healing (highest PLR of 1.58) and nonhealing (highest NLR of 0.28).^31,34^ The previously mentioned study by Elghazaly et al. reported an insufficient diagnostic performance of the TBI when a cut-off value of 0.8 was applied (PLR 2.4 and NLR 0.63).

Toe pressure was assessed in eight studies.^27,31,35,38,40,43,44,47^ Different thresholds for absolute blood pressure were used (15, 20, 30, 40, 45, and 54 mmHg), whereas a toe pressure of 30 mmHg was most frequently applied in four studies.^38,40,44,47^ Holstein et al. showed an infinite PLR at this threshold, followed by Kalani et al. (PLR 5.0).^40,44^ The lowest NLR (0.28) was found also by Holstein et al., indicating a small effect on the ability to exclude wound healing.^40^ When looking at the two studies that used a lower cut-off value (15 and 20 mmHg), lower NLRs were achieved (0.1 and 0.1).^27,43^ This suggests a better capability of predicting nonhealing of an ulcer, which is in accordance with the theoretical background of the test. If higher cut-off values for the toe pressure were used (40, 45, and 54 mmHg), no clear conclusions could be made regarding the diagnostic performance, as both lower and higher PLRs and NLRs were found (Table 3).^27,31,35,44^

#### Transcutaneous oxygen pressure

Thirteen studies looked at TcPO_2_ as a predictor for ulcer healing.^23,26,29,31–35,42,44–46,48^ The majority of these studies (seven) applied a cut-off value between 25 and 32 mmHg, whereas, respectively, three^23,32,45^ and four^31,33,42,48^ studies looked at thresholds below and above these values. In general, a highly varied diagnostic performance was seen. Four studies showed high prognostic accuracies with a reasonable trade-off between predicting ulcer healing (PLR > 5) and nonhealing (NLR < 0.2).^26,34,44,45^ Except for Karanfilian et al. (TcPO_2_ 10 mmHg), these studies used a cut-off value between 25 and 32 mmHg. However, according to Table 2 (QUIPS-tool), major concerns were seen in all these studies regarding their potential risk of bias with small patient samples and heterogeneous groups. The other four studies that looked at a threshold between 25 and 32 mmHg showed poor prognostic performances on predicting both healing and nonhealing of ulcers (Table 3). Hypothetically, higher cut-off values of TcPO_2_ should be more able to predict ulcer healing. However, the studies that investigated these values (40 and 43 mmHg) were not able to prove this.^31,33,42,48^ Conversely, in the studies that applied lower thresholds, slightly lower NLRs were found (0.43, 0.58, and 0).^23,32,45^ An overview of PLR and NLR ranges can be found in Table 3.

#### Other bedside tests

Skin perfusion pressure (SPP) was investigated by three studies, all of which applied different thresholds (30, 40, and 43 mmHg).^22,39,40^ Holstein et al. studied a cut-off value of 30 mmHg, leading to a good PLR of 6.4 but an insufficient NLR of 0.40. A threshold of 40 mmHg was applied by Faris et al., which showed a PLR of 4.9 and a NLR of 0.04.^39^ Although these diagnostic performances are decent, the low number of patients (*n* = 61), unclear design, and outcome definition (healing after local amputation was regarded as healing as well) led to a high risk of bias. Kawai et al. used a cut-off value of 43 mmHg, which resulted in a PLR of 11.7 and a NLR of 0.35.^22^

The presence of peripheral palpable pulses with respect to ulcer healing was examined by two studies.^28,29^ Apelqvist et al. looked at pulses in the femoral, popliteal, and pedal arteries, but could not find any prognostic relevance (all PLRs below 3 and NLRs above 0.4).^28^ Ballard et al. investigated the presence of pedal pulses, which led to a PLR of 5.5 and a NLR of 0.45.^29^

The skin hydration level (SHL) was studied in two separate articles by the same author.^48,49^ Both studies led to a poor prognostic performance with a PLR of 1.3/2.4 and NLR of 0.72/0.40, respectively.

Three studies explored bedside tests that were not mentioned in other literature. Elghazaly et al. looked at monophasic or absent waveforms with the podiatry ankle (PAD) scan, which revealed a PLR of 1.29 and NLR of 0.56.^31^ The ankle peak systolic velocity (APSV) was studied by Bishara et al., with a corresponding PLR of 9.88 and NLR of 0.02.^30^ Karanfilian et al. investigated the laser Doppler velocimetry (LDV) at a threshold of 40 mV, which led to a PLR of 14.9 and NLR of 0.13.^45^

### Healing of minor amputation wounds

In total, eight studies looked at wound healing after minor amputations.^25,50–56^

#### ABI and ankle pressure

Ankle pressure was investigated most frequently as a bedside test after amputation.^50–53,55^ Only two of the five studies applied the same threshold (60 mmHg).^53,55^ Other included cut-off values were 40, 50, 70, and 75 mmHg. Except for Holstein et al.,^51^ which revealed a NLR of 0.0 (and PLR of 1.1) at a threshold of 50 mmHg, none of the studies was able to show a reliable prognostic performance regarding amputation healing (all PLRs below 5 and NLRs above 0.1). However, this study had a high risk of bias according to the QUIPS-tool (Table 2) due to an unclear definition of healing, statistical analysis (no prespecified cut-off value), and participation.

Two studies looked at the ABI, which showed fairly good results regarding nonhealing (NLRs of 0.12 and 0.20), but were insufficient in predicting wound healing after minor amputation (PLRs of 2.0 and 4.6) when using cut-off values of 0.5 and 0.7.^52,56^

#### Toe–brachial index and toe pressure

Toe pressure was explored in three studies.^51,52,54^ All of these used different cut-off values for predicting amputation healing (15, 30, and 38 mmHg). Both Larsson et al. and Vitti et al. showed low NLRs of 0.10 and 0 when applying a threshold of 15 and 38 mmHg, respectively.^52,54^ However, corresponding PLRs were inadequate (1.7 and 2.3), and the quality assessment revealed a high risk of bias for both studies (Table 2). The third study by Holstein et al. applied a threshold of 30 mmHg, which showed a poor prognostic performance (PLR 1.3 and NLR 0.7).^51^ Only one study by Larsson et al. investigated the TBI, which resulted in a PLR of 1.9 and NLR of 0.09 when using a cut-off value of 0.10.^52^

#### Other measures

Wyss et al. studied TcPO_2_ (> 31 mmHg), which led to a fairly good prognostic performance (PLR 5.9 and NLR 0.2) in 26 foot or Syme amputations.^25^ Many major confounders were not assessed or reported in this study. Pulse volume recordings were investigated by Gibbons et al., showing a PLR of 1.8 and NLR of 0.18.^50^ One study investigated the skin blood flow at 12 and 16 mL 100 g^−1^ min^−1^, revealing an infinite high PLR, as all wounds healed above the chosen cut-off values.^55^ Corresponding NLRs were 0.5 and 0.7, respectively.

## Discussion

This systematic review evaluated 35 studies investigating the prognostic performance of bedside tests for primary ulcer and minor amputation wound healing in patients prone to MAC. None of the studied bedside tests was able to show reliable prognostic performances for healing of primary ulcers or minor amputation wounds, indicated by insufficient trade-offs between PLRs and NLRs at different thresholds. This emphasizes the need for better prognostic tools to evaluate wound healing in these patient groups.

The best prognostic performance of the different bedside tests was seen with TcPO_2_, particularly in predicting failure of healing. As can be found in Table 3, NLR ranges between 0 and 0.58 were present when applying a threshold around 30 mmHg. Of seven studies that used this cut-off value, five showed a NLR below 0.2 (indicating a moderate to large effect on the ability to rule out ulcer healing). However, only two of these studies had a PLR above 5.0, thus providing an inadequate trade-off for predicting ulcer healing in a clinical setting. From these results, it can be deducted that in patients prone to MAC and a low TcPO_2_ measurement, no wait-and-see approach should be applied. Another commonly used bedside test in current clinical practice is the toe pressure. As digital arteries theoretically should be less susceptible for MAC, it is hypothesized that its diagnostic and prognostic performance should be better (than AP/ABI) in diabetic patients.^15^ Ten studies investigated toe pressure, where a pressure of 30 mmHg was most frequently used as the cut-off value. This is, to the best of our knowledge, also a threshold that is frequently mentioned in daily practice. Nevertheless, none of those studies provided a reasonable trade-off between proving and excluding ulcer healing. All NLRs were above 0.2, suggesting an inadequate performance to predict nonhealing. Furthermore, for predicting wound healing, no TP threshold was suitable. The same results were seen for the TBI.

Most of the studies included were of poor to moderate methodological quality. This was mainly caused by the use of unclear definitions in wound healing, use of unclear classification systems regarding ulcer severity, and heterogeneous study populations without adequate subgroup analyses. Only two of the included studies had a low risk of bias according to the QUIPS-2 tool.^30,31^ Elghazaly et al. investigated ABI, ankle pressure, TBI, toe pressure, and TcPO_2_, but none showed accurate prognostic performance. The APSV was investigated by Bishara et al., resulting in a high PLR (9.9) and low NLR (0.02), indicating a promising accuracy potentially applicable in daily practice.^30^ Unfortunately, this was the only article studying the APSV, limiting its generalizability.

The insufficient prognostic performances of bedside tests to predict ulcer healing in patients prone to MAC are unsurprising, as it is well known that these ulcers have a complex multifactorial etiology. Moreover, most of the bedside tests merely assess macrovascular perfusion, which is just one of the important components in ulcer healing. MAC likely limits the reliability of macrovascular perfusion assessment (ABI, AP, TBI, TP, palpable pulses) because it can cause falsely elevated blood pressure measurements, hampering prognostic capabilities. Furthermore, microvascular perfusion, wound characteristics, and infection also play major roles in ulcer healing. The currently used prognostic bedside tests do not seem to sufficiently address all these factors, and the absence of macrovascular insufficiency does not necessarily mean that an ulcer will heal.

All included studies in this review investigated ulcer or amputation healing in patients prone to MAC. However, almost all studies only investigated patients with DM. CKD and aging were not studied specifically but are well known to increase the likelihood of MAC.^7^ Moreover, although falsely normal values can occur, the presence of MAC is often characterized by an ABI > 1.3. Unfortunately, no studies reported subgroup analyses for these values.^8–10^ Only one study investigated healing in a subgroup of patients with chronic renal failure (CRF) using a cut-off value of 1.1 for ABI, demonstrating inadequate predictive capabilities (PLR 1.0, NLR 0.98).^23^ Because patients receiving hemodialysis for end-stage CKD often develop leg ulcers, it is quite puzzling so little is known about this patient population and ulcer healing. The lack of evidence regarding the predictive capabilities of bedside tests concerning wound healing in patients with CKD, advanced age, or ABI > 1.3 limits the generalizability of the results of this review for the entire MAC population.

When comparing our review with previous literature, particularly the studies by Chuter et al. and Forsythe et al., we observe both similarities and differences.^57,58^ Though these articles focused specifically on diabetic foot ulcer healing, our study broadened the scope to include all patients prone to MAC. Our analysis revealed that most existing research clusters on diabetic patients, and little is known about ulcer healing in patients with CKD or older age. Nonetheless, it is crucial to emphasize the limited understanding of prognostic bedside tests available for these patient groups. This indicates a significant knowledge gap that warrants further investigation. Moreover, our search strategy successfully identified additional relevant articles that contribute to this topic.

To improve future research, various issues encountered in this review should be addressed. First and foremost, the definition of wound healing needs to be clearly described and preferably used uniformly across the research field. Second, as a continuation, a standardized classification system of ulcer severity should be used in order to improve applicability in daily practice and comparability of results between studies. This systematic review also highlights the significant heterogeneity of wounds across the included studies, with classification systems being either variable or absent. Current guidelines advise using the Wound, Ischemia, and foot Infection (WIfI) classification system to define ulcer severity and characteristics as this is proven to correlate with the probability of wound healing.^59–61^ Third, the majority of articles in this review were retrospective cohort studies, introducing multiple risks of bias. Among others, selection bias and the lack of consistent and complete data gathering (dependent on parameters and outcomes recorded in patient files) could have occurred. Future studies should aim to be prospective, incorporate a clear definition of ulcer healing, use a standardized wound classification system, and minimize heterogeneity by separating patients based on underlying etiology. Moreover, these studies could also explore the predictive value of combining multiple tests within a prediction model.

Finally, we believe that new statistical tools could help in better assessment of the predictive capabilities of ulcer or amputation healing. An important limitation of current parameters (sensitivity, specificity, and PLR/NLR) is that only information at one threshold based on data from an entire cohort is provided. In the near future, predictive models (e.g., multistate modeling) could be used to evaluate the chances of important clinical outcomes (ulcer healing, amputation, and mortality).^62^ A distinct advantage of these models is that they can incorporate individual patient and wound characteristics. Because wound healing is a complex and multifactorial problem, an extensive overview of multiple patient characteristics is needed for accurate prediction. Predictive models can incorporate multiple relevant variables for individual patients, including local wound characteristics, (intraoperative) macrovascular assessment, and microvascular perfusion (e.g., near-infrared fluorescence or hyperspectral imaging) to provide a tailor-made prognosis.^63,64^ The rise and development of artificial intelligence might aid in creating these tools as well.

## Conclusion

This review demonstrates that none of the currently used bedside tests has an acceptable prognostic performance for ulcer healing in patients prone to MAC. The methodological quality of the included studies in this review raised serious concerns on several important domains, leading to a high risk of bias. Future prospective studies should aim to incorporate a clear definition of ulcer healing, use a standardized wound classification system, and minimize heterogeneity by separating patients based on the underlying etiology of their ulcer. A combined assessment of microvascular and macrovascular perfusion status could improve the prediction of wound healing.

## Supplemental Material

sj-pdf-1-vmj-10.1177_1358863X241309326 – Supplemental material for Prognostic performance of bedside tests for predicting ulcer healing and wound healing after minor amputation in patients prone to medial arterial calcification: A systematic review Supplemental material, sj-pdf-1-vmj-10.1177_1358863X241309326 for Prognostic performance of bedside tests for predicting ulcer healing and wound healing after minor amputation in patients prone to medial arterial calcification: A systematic review by Siem A Willems, Jelle A Nieuwstraten, Abbey Schepers, Jan van Schaik, Pim van den Hoven, Joost R van der Vorst, Jaap F Hamming and Jeroen JWM Brouwers in Vascular Medicine

sj-pdf-2-vmj-10.1177_1358863X241309326 – Supplemental material for Prognostic performance of bedside tests for predicting ulcer healing and wound healing after minor amputation in patients prone to medial arterial calcification: A systematic review Supplemental material, sj-pdf-2-vmj-10.1177_1358863X241309326 for Prognostic performance of bedside tests for predicting ulcer healing and wound healing after minor amputation in patients prone to medial arterial calcification: A systematic review by Siem A Willems, Jelle A Nieuwstraten, Abbey Schepers, Jan van Schaik, Pim van den Hoven, Joost R van der Vorst, Jaap F Hamming and Jeroen JWM Brouwers in Vascular Medicine
