# Supplementary material for: Prognostic performance of bedside tests for predicting ulcer healing and wound healing after minor amputation in patients prone to medial arterial calcification: A systematic review
Source: Vasc Med. 2025 Jan 21;30(2):250–60. doi: 10.1177/1358863X241309326 (PMC12014953; doi:10.1177/1358863X241309326)
Supplement: sj-pdf-1-vmj-10.1177_1358863X241309326 – Supplemental material for Prognostic performance of bedside tests for predicting ulcer healing and wound healing after minor amputation in patients prone to medial arterial calcification: A systematic review [file sj-pdf-1-vmj-10.1177_1358863X241309326.pdf]

Supplement S1: Search strategy

In cooperation with a trained librarian (JWS), a detailed search strategy was composed. The following databases were searched: PubMed, Embase (OVID-version), Web of Science, Cochrane Library, and Emcare. The query consisted of the combination of the following concepts:

- Predicting wound healing
- Bedside non-invasive diagnostic tests
- Patients prone to medial arterial calcification

These concepts were combined using two search strands in order to maximize relevancy and minimize noise. For the different concepts, all relevant keyword variations were used, not only keyword variations in the controlled vocabularies of the various databases, but the free text word variations of these concepts as well. The search strategy was optimized for all consulted databases, taking into account the differences of the various controlled vocabularies as well as the differences of database-specific technical variations (e.g., the use of quotation marks). The search was limited to English language articles. The final search was performed on June 20th, 2023. The bibliographic databases yielded 943 references. Full details of the search strategy can be found in Appendix A (see below).

Appendix A - Search strategy details

Database	Search Strategy	Number of references	Number of unique references
PubMed	((("Peripheral Arterial Disease"[majr] OR "Peripheral Arterial Disease"[tiab] OR "Peripheral Arterial Diseases"[tiab] OR "Peripheral Artery Disease"[tiab] OR "Peripheral Artery Diseases"[tiab] OR "Peripheral Arterial Disorder"[tiab] OR "Peripheral Arterial Disorders"[tiab] OR "Peripheral Artery Disorder"[tiab] OR "Peripheral Artery Disorders"[title/abstract:~2] OR ("PAD"[tiab] AND ("artery"[tiab] OR "arteries"[tiab] OR "arterial"[tiab])) OR "peripheral arterial occlusive disease"[tiab] OR "peripheral arterial occlusive diseases"[tiab] OR "peripheral artery occlusive disease"[tiab] OR "peripheral artery occlusive diseases"[tiab] OR "Peripheral Vascular Diseases"[majr:noexp] OR "Peripheral Vascular Disease"[tiab] OR "Peripheral Vascular Diseases"[tiab] OR "Peripheral Vascular Disorder"[tiab] OR "Peripheral Vascular Disorders"[tiab] OR "Peripheral Angiopathies"[tiab] OR "Peripheral Angiopathy"[tiab] OR "Peripheral Arteriopathies"[tiab] OR "Peripheral Arteriopathy"[tiab] OR "Peripheral Vasculopathies"[tiab] OR "Peripheral Vasculopathy"[tiab] OR "Monckeberg Medial Calcific Sclerosis"[majr] OR "Monckeberg's Medial Calcific Sclerosis"[tiab] OR "Monckeberg's Sclerosis"[tiab] OR "Monckeberg Sclerosis"[tiab] OR "Monckebergs Sclerosis"[title/abstract:~3] OR "Mönckeberg's Medial Calcific Sclerosis"[tiab] OR "Mönckeberg's Sclerosis"[tiab] OR "Mönckeberg Sclerosis"[tiab] OR "Mönckebergs Sclerosis"[title/abstract:~3] OR "Monckeberg"[tiab] OR "Monckeberg*"[tiab] OR "Moenckeberg"[tiab] OR "Moenckeberg*"[tiab] OR "Medial Calcific Sclerosis"[tiab] OR "Medial Calcific Scleroses"[title/abstract:~3] OR "Mönckeberg Medial Calcific Sclerosis"[tiab] OR "incompressible arter*"[tiab] OR "incompressible vessel*"[tiab] OR "medial calcified artery"[title/abstract:~3] OR "medial calcified arteries"[title/abstract:~3] OR (("Arterial Occlusive Diseases"[majr] OR "Peripheral Vascular Diseases"[majr]) AND ("peripheral"[tiab] OR "peripheral*"[tiab] OR "limbs"[tiab] OR "limb"[tiab] OR "leg"[tiab] OR "legs"[tiab] OR "extremity"[tiab] OR "extremities"[tiab] OR "arm"[tiab] OR "arms"[tiab])) OR "Intermittent Claudication"[majr] OR "Intermittent Claudication"[tiab] OR "Leriche Syndrome"[majr] OR "Leriche Syndrome"[tiab] OR "Leriche's Syndrome"[tiab] OR "Leriches Syndrome"[tiab] OR "Arteriosclerosis Obliterans"[majr] OR "Arteriosclerosis Obliterans"[tiab] OR (("Atherosclerosis"[majr] OR "Arteriosclerosis"[majr] OR "Atherosclerosis"[tiab] OR "Arteriosclerosis"[tiab]) AND ("peripheral"[tiab] OR "peripheral*"[tiab] OR "limbs"[tiab] OR "limb"[tiab] OR "leg"[tiab] OR "legs"[tiab] OR "extremity"[tiab] OR "extremities"[tiab] OR "arm"[tiab] OR "arms"[tiab])) OR "Fontaine IV"[tiab] OR "Diabetic Foot"[majr] OR "Diabetic Foot"[tiab] OR "Diabetic Feet"[tiab] OR (("Diabetes Mellitus"[majr] OR "diabetes"[tiab] OR "diabet*"[tiab] OR "Renal Insufficiency, Chronic"[majr] OR "Chronic Kidney Disease"[tiab] OR "Chronic Kidney Diseases"[tiab] OR "Chronic Kidney Failure"[tiab] OR "Chronic Kidney Insufficiency"[tiab] OR "Chronic Renal Disease"[tiab] OR "Chronic Renal Diseases"[tiab] OR "Chronic Renal Failure"[tiab] OR "Chronic Renal Insufficiency"[tiab] OR "End Stage Kidney Disease"[tiab] OR "End Stage Renal Disease"[tiab] OR "End-Stage Renal Failure"[tiab] OR "End-Stage Kidney Failure"[tiab] OR "ESRD"[tiab]) AND ("peripheral"[tiab] OR "peripheral*"[tiab] OR "Extremities"[majr] OR "Foot Diseases"[majr] OR "limbs"[tiab] OR "limb"[tiab] OR "leg"[tiab] OR "legs"[tiab] OR "extremity"[tiab] OR "extremities"[tiab] OR "arm"[tiab] OR "arms"[tiab] OR "foot"[tiab] OR "feet"[tiab] OR "toe"[tiab] OR "toes"[tiab] OR "finger"[tiab] OR "fingers"[tiab]))) AND ("Ankle Brachial Index"[mesh] OR "Ankle Brachial Indices"[tw] OR "Ankle-Brachial Index"[tw] OR "Ankle-Brachial Indices"[tw] OR "anklebrachial index"[tw] OR "anklebrachialindex"[tw] OR "ankle brachial"[tw] OR "anklebrachial"[tw] OR "ABI"[tw] OR "Toe Brachial Index"[tw] OR "Toe Brachial Indices"[tw] OR "Toe-Brachial Index"[tw] OR "Toe-Brachial Indices"[tw] OR "Toe Pressure"[tw] OR "toe brachial"[tw] OR "toebrachial"[tw] OR "TBI"[tw] OR "TP"[tw] OR "Oximetry"[mesh] OR "Oximetry"[tw] OR "Oximetries"[tw] OR "Oximetr*"[tw] OR "Pulse Oximetries"[tw] OR "Pulse Oximetry"[tw] OR "transcutaneous oxygen tension"[tw] OR "transcutaneous oxygen"[tw] OR "Pulse"[mesh] OR "Pulse*"[tw] OR "pulsation"[tw] OR "pulsations"[tw] OR "Blood Gas Monitoring, Transcutaneous"[mesh] OR "Transcutaneous Blood Gas Monitoring"[tw] OR "Transcutaneous Capnometry"[tw] OR "PtcO2"[tw] OR "TcPCO2"[tw] OR "Doppler waveform"[tw] OR "Doppler waveforms"[tw] OR "Doppler wave form"[tw] OR "Doppler wave forms"[tw] OR "Ultrasonography, Doppler, Pulsed"[mesh:noexp] OR "Pulsed Doppler"[tw] OR "Doppler Pulsed"[tw] OR "non-invasive"[tw] OR "non-invasiv*"[tw] OR "point-of-care test"[tw] OR "point-of-care tests"[tw] OR "Ankle Pressure"[tw] OR "Ankle Pressures"[tw] OR "Skin Temperature"[Mesh] OR "Skin Temperature"[tw] OR "Skin Temperatures"[tw] OR "Blood Pressure Determination"[Mesh] OR "blood pressure measurement"[tw] OR "blood pressure measurements"[tw]) AND ("Wound Healing"[Mesh] OR "Wound Healing"[tw] OR "Wound Healing"[title/abstract:~4] OR "Wound Heal"[title/abstract:~4] OR "Wound Healed"[title/abstract:~4] OR "Wound Heals"[title/abstract:~4] OR "Wounds Healing"[tw] OR "Wounds Healing"[title/abstract:~4] OR "Wounds Heal"[title/abstract:~4] OR "Wounds Healed"[title/abstract:~4] OR "Wounds Heals"[title/abstract:~4] OR "Ulcer Healing"[tw] OR "Ulcer Healing"[title/abstract:~4] OR "Ulcer Heal"[title/abstract:~4] OR "Ulcer Healed"[title/abstract:~4] OR "Ulcer Heals"[title/abstract:~4] OR "Ulcers Healing"[tw] OR "Ulcers Healing"[title/abstract:~4] OR "Ulcers Heal"[title/abstract:~4] OR "Ulcers Healed"[title/abstract:~4] OR "Ulcers Heals"[title/abstract:~4] OR (("predictor"[tw] OR "predictors"[tw] OR "predictive factor"[tw] OR "predictive factors"[tw] OR "predicting"[tw] OR "Forecasting"[mesh]) AND ("Amputation, Surgical"[Mesh] OR "Amputation"[tw])) OR "Wound Outcome"[title/abstract:~4] OR "Wound Outcomes"[title/abstract:~4] OR "Wounds Outcome"[title/abstract:~4] OR "Wounds Outcomes"[title/abstract:~4] OR "Ulcer Outcome"[tw] OR	711	711

	"Ulcer Outcome"[title/abstract:~4] OR "Ulcer Outcomes"[title/abstract:~4] OR "Ulcers Outcome"[tw] OR "Ulcers Outcome"[title/abstract:~4] OR "Ulcers Outcomes"[title/abstract:~4]) AND ("Sensitivity and Specificity"[mesh] OR "Sensitivity"[tiab] OR "Specificity"[tiab] OR "Predictive Value"[tiab] OR "ROC Curve"[tiab] OR "Signal-To-Noise"[tiab] OR "limit of detection"[tiab] OR "Cohort Studies"[mesh] OR "Follow-Up Studies"[mesh] OR "Longitudinal Studies"[mesh] OR "Prospective Studies"[mesh] OR "Retrospective Studies"[mesh] OR "Cohort"[tiab] OR "Cohorts"[tiab] OR "Follow-Up"[tiab] OR "Longitudinal"[tiab] OR "Prospective"[tiab] OR "Retrospective"[tiab] OR "Cross-Sectional Studies"[mesh] OR "Cross-Sectional"[tiab] OR "Observational Study"[pt] OR "Observational Studies as Topic"[mesh] OR "Observational Study"[tiab] OR "effectiveness"[tiab] OR "efficacy"[tiab])) **OR** (("Peripheral Arterial Disease"[majr] OR "Peripheral Arterial Disease"[tiab] OR "Peripheral Arterial Diseases"[tiab] OR "Peripheral Artery Disease"[tiab] OR "Peripheral Artery Diseases"[tiab] OR "Peripheral Arterial Disorder"[tiab] OR "Peripheral Arterial Disorders"[tiab] OR "Peripheral Artery Disorder"[tiab] OR "Peripheral Artery Disorders"[title:~2] OR ("PAD"[tiab] AND ("artery"[tiab] OR "arteries"[tiab] OR "arterial"[tiab])) OR "peripheral arterial occlusive disease"[tiab] OR "peripheral arterial occlusive diseases"[tiab] OR "peripheral artery occlusive disease"[tiab] OR "peripheral artery occlusive diseases"[tiab] OR "Peripheral Vascular Diseases"[majr:noexp] OR "Peripheral Vascular Disease"[tiab] OR "Peripheral Vascular Diseases"[tiab] OR "Peripheral Vascular Disorder"[tiab] OR "Peripheral Vascular Disorders"[tiab] OR "Peripheral Angiopathies"[tiab] OR "Peripheral Angiopathy"[tiab] OR "Peripheral Arteriopathies"[tiab] OR "Peripheral Arteriopathy"[tiab] OR "Peripheral Vasculopathies"[tiab] OR "Peripheral Vasculopathy"[tiab] OR "Monckeberg Medial Calcific Sclerosis"[majr] OR "Monckeberg's Medial Calcific Sclerosis"[tiab] OR "Monckeberg's Sclerosis"[tiab] OR "Monckeberg Sclerosis"[tiab] OR "Monckebergs Sclerosis"[title:~3] OR "Mönckeberg's Medial Calcific Sclerosis"[tiab] OR "Mönckeberg's Sclerosis"[tiab] OR "Mönckeberg Sclerosis"[tiab] OR "Mönckebergs Sclerosis"[title:~3] OR "Monckeberg"[tiab] OR "Monckeberg*"[tiab] OR "Moenckeberg"[tiab] OR "Moenckeberg*"[tiab] OR "Medial Calcific Sclerosis"[tiab] OR "Medial Calcific Scleroses"[title:~3] OR "Mönckeberg Medial Calcific Sclerosis"[tiab] OR "incompressible arter*"[tiab] OR "incompressible vessel*"[tiab] OR "medial calcified artery"[title:~3] OR "medial calcified arteries"[title:~3] OR (("Arterial Occlusive Diseases"[majr] OR "Peripheral Vascular Diseases"[majr]) AND ("peripheral"[tiab] OR "peripheral*"[tiab] OR "limbs"[tiab] OR "limb"[tiab] OR "leg"[tiab] OR "legs"[tiab] OR "extremity"[tiab] OR "extremities"[tiab] OR "arm"[tiab] OR "arms"[tiab])) OR "Intermittent Claudication"[majr] OR "Intermittent Claudication"[tiab] OR "Leriche Syndrome"[majr] OR "Leriche Syndrome"[tiab] OR "Leriche's Syndrome"[tiab] OR "Leriches Syndrome"[tiab] OR "Arteriosclerosis Obliterans"[majr] OR "Arteriosclerosis Obliterans"[tiab] OR (("Atherosclerosis"[majr] OR "Arteriosclerosis"[majr] OR "Atherosclerosis"[tiab] OR "Arteriosclerosis"[tiab]) AND ("peripheral"[tiab] OR "peripheral*"[tiab] OR "limbs"[tiab] OR "limb"[tiab] OR "leg"[tiab] OR "legs"[tiab] OR "extremity"[tiab] OR "extremities"[tiab] OR "arm"[tiab] OR "arms"[tiab])) OR "Fontaine IV"[tiab] OR "Diabetic Foot"[majr] OR "Diabetic Foot"[tiab] OR "Diabetic Feet"[tiab] OR (("Diabetes Mellitus"[majr] OR "diabetes"[tiab] OR "diabet*"[tiab] OR "Renal Insufficiency, Chronic"[majr] OR "Chronic Kidney Disease"[tiab] OR "Chronic Kidney Diseases"[tiab] OR "Chronic Kidney Failure"[tiab] OR "Chronic Kidney Insufficiency"[tiab] OR "Chronic Renal Disease"[tiab] OR "Chronic Renal Diseases"[tiab] OR "Chronic Renal Failure"[tiab] OR "Chronic Renal Insufficiency"[tiab] OR "End Stage Kidney Disease"[tiab] OR "End Stage Renal Disease"[tiab] OR "End-Stage Renal Failure"[tiab] OR "End-Stage Kidney Failure"[tiab] OR "ESRD"[tiab]) AND ("peripheral"[tiab] OR "peripheral*"[tiab] OR "Extremities"[majr] OR "Foot Diseases"[majr] OR "limbs"[tiab] OR "limb"[tiab] OR "leg"[tiab] OR "legs"[tiab] OR "extremity"[tiab] OR "extremities"[tiab] OR "arm"[tiab] OR "arms"[tiab] OR "foot"[tiab] OR "feet"[tiab] OR "toe"[tiab] OR "toes"[tiab] OR "finger"[tiab] OR "fingers"[tiab]))) AND ("Ankle Brachial Index"[mesh] OR "Ankle Brachial Indices"[tw] OR "Ankle-Brachial Index"[tw] OR "Ankle-Brachial Indices"[tw] OR "anklebrachial index"[tw] OR "anklebrachialindex"[tw] OR "ankle brachial"[tw] OR "anklebrachial"[tw] OR "ABI"[tw] OR "Toe Brachial Index"[tw] OR "Toe Brachial Indices"[tw] OR "Toe-Brachial Index"[tw] OR "Toe-Brachial Indices"[tw] OR "Toe Pressure"[tw] OR "toe brachial"[tw] OR "toebrachial"[tw] OR "TBI"[tw] OR "TP"[tw] OR "Oximetry"[mesh] OR "Oximetry"[tw] OR "Oximetries"[tw] OR "Oximetr*"[tw] OR "Pulse Oximetries"[tw] OR "Pulse Oximetry"[tw] OR "transcutaneous oxygen tension"[tw] OR "transcutaneous oxygen"[tw] OR "Pulse"[mesh] OR "Pulse*"[tw] OR "pulsation"[tw] OR "pulsations"[tw] OR "Blood Gas Monitoring, Transcutaneous"[mesh] OR "Transcutaneous Blood Gas Monitoring"[tw] OR "Transcutaneous Capnometry"[tw] OR "PtcO2"[tw] OR "TcPCO2"[tw] OR "Doppler waveform"[tw] OR "Doppler waveforms"[tw] OR "Doppler wave form"[tw] OR "Doppler wave forms"[tw] OR "Ultrasonography, Doppler, Pulsed"[mesh:noexp] OR "Pulsed Doppler"[tw] OR "Doppler Pulsed"[tw] OR "non-invasive"[tw] OR "non-invasiv*"[tw] OR "point-of-care test"[tw] OR "point-of-care tests"[tw] OR "Ankle Pressure"[tw] OR "Ankle Pressures"[tw] OR "Skin Temperature"[Mesh] OR "Skin Temperature"[tw] OR "Skin Temperatures"[tw] OR "Blood Pressure Determination"[Mesh] OR "blood pressure measurement"[tw] OR "blood pressure measurements"[tw]) AND ("Wound Healing"[majr] OR "Wound Healing"[ti] OR "Wound Healing"[title:~4] OR "Wound Heal"[title:~4] OR "Wound Healed"[title:~4] OR "Wound Heals"[title:~4] OR "Wounds Healing"[ti] OR "Wounds Healing"[title:~4] OR "Wounds Heal"[title:~4] OR "Wounds Healed"[title:~4] OR "Wounds Heals"[title:~4] OR "Ulcer Healing"[ti] OR "Ulcer Healing"[title:~4] OR "Ulcer Heal"[title:~4] OR "Ulcer Healed"[title:~4] OR "Ulcer Heals"[title:~4] OR "Ulcers Healing"[ti] OR "Ulcers Healing"[title:~4] OR "Ulcers Heal"[title:~4] OR "Ulcers Healed"[title:~4] OR "Ulcers Heals"[title:~4] OR (("predictor"[ti] OR "predictors"[ti] OR "predictive factor"[ti] OR "predictive factors"[ti] OR "predicting"[ti] OR "Forecasting"[majr]) AND ("Amputation, Surgical"[majr] OR "Amputation"[ti])) OR "Wound Outcome"[title:~4] OR "Wound Outcomes"[title:~4] OR "Wounds Outcome"[title:~4] OR "Wounds Outcomes"[title:~4] OR "Ulcer		

	Outcome"[ti] OR "Ulcer Outcome"[title:~4] OR "Ulcer Outcomes"[title:~4] OR "Ulcers Outcome"[ti] OR "Ulcers Outcome"[title:~4] OR "Ulcers Outcomes"[title:~4])))		
Embase (OVID-version)	(((*"Peripheral Occlusive Artery Disease"/ OR "Peripheral Arterial Disease".ti,ab OR "Peripheral Arterial Diseases".ti,ab OR "Peripheral Artery Disease".ti,ab OR "Peripheral Artery Diseases".ti,ab OR "Peripheral Arterial Disorder".ti,ab OR "Peripheral Arterial Disorders".ti,ab OR "Peripheral Artery Disorder".ti,ab OR ("PAD".ti,ab AND ("artery".ti,ab OR "arteries".ti,ab OR "arterial".ti,ab)) OR "peripheral arterial occlusive disease".ti,ab OR "peripheral arterial occlusive diseases".ti,ab OR "peripheral artery occlusive disease".ti,ab OR "peripheral artery occlusive diseases".ti,ab OR "Peripheral Vascular Disease"/ OR "Peripheral Vascular Disease".ti,ab OR "Peripheral Vascular Diseases".ti,ab OR "Peripheral Vascular Disorder".ti,ab OR "Peripheral Vascular Disorders".ti,ab OR "Peripheral Angiopathies".ti,ab OR "Peripheral Angiopathy".ti,ab OR "Peripheral Arteriopathies".ti,ab OR "Peripheral Arteriopathy".ti,ab OR "Peripheral Vasculopathies".ti,ab OR "Peripheral Vasculopathy".ti,ab OR *"Monckeberg medial calcific sclerosis"/ OR "Monckeberg's Medial Calcific Sclerosis".ti,ab OR "Monckeberg's Sclerosis".ti,ab OR "Monckeberg Sclerosis".ti,ab OR ("Monckeberg*" ADJ3 "Sclerosis").ti,ab OR "Mönckeberg's Medial Calcific Sclerosis".ti,ab OR "Mönckeberg's Sclerosis".ti,ab OR "Mönckeberg Sclerosis".ti,ab OR ("Mönckeberg*" ADJ3 "Sclerosis").ti,ab OR "Monckeberg".ti,ab OR "Monckeberg*".ti,ab OR "Moenckeberg".ti,ab OR "Moenckeberg*".ti,ab OR "Medial Calcific Sclerosis".ti,ab OR ("Medial" ADJ3 "Calcific" ADJ3 "Scleroses").ti,ab OR "Mönckeberg Medial Calcific Sclerosis".ti,ab OR "incompressible arter*".ti,ab OR "incompressible vessel*".ti,ab OR ("medial" ADJ3 "calcified" ADJ3 "artery").ti,ab OR ("medial" ADJ3 "calcified" ADJ3 "arteries").ti,ab OR *"Intermittent Claudication"/ OR "Intermittent Claudication".ti,ab OR "Leriche Syndrome"/ OR "Leriche Syndrome".ti,ab OR "Leriche's Syndrome".ti,ab OR "Leriches Syndrome".ti,ab OR "Arteriosclerosis Obliterans"/ OR "Arteriosclerosis Obliterans".ti,ab OR ((exp *"Atherosclerosis"/ OR exp *"Arteriosclerosis"/ OR "Atherosclerosis".ti,ab OR "Arteriosclerosis".ti,ab) AND ("peripheral".ti,ab OR "peripheral*".ti,ab OR "limbs".ti,ab OR "limb".ti,ab OR "leg".ti,ab OR "legs".ti,ab OR "extremity".ti,ab OR "extremities".ti,ab OR "arm".ti,ab OR "arms".ti,ab)) OR "Fontaine IV".ti,ab OR *"Diabetic Foot"/ OR "Diabetic Foot".ti,ab OR "Diabetic Feet".ti,ab OR ((exp *"Diabetes Mellitus"/ OR "diabetes".ti,ab OR "diabet*".ti,ab OR exp *"Chronic Kidney Failure"/ OR "Chronic Kidney Disease".ti,ab OR "Chronic Kidney Diseases".ti,ab OR "Chronic Kidney Failure".ti,ab OR "Chronic Kidney Insufficiency".ti,ab OR "Chronic Renal Disease".ti,ab OR "Chronic Renal Diseases".ti,ab OR "Chronic Renal Failure".ti,ab OR "Chronic Renal Insufficiency".ti,ab OR "End Stage Kidney Disease".ti,ab OR "end stage renal disease"/ OR "End Stage Renal Disease".ti,ab OR "End-Stage Renal Failure".ti,ab OR "End-Stage Kidney Failure".ti,ab OR "ESRD".ti,ab) AND ("peripheral".ti,ab OR "peripheral*".ti,ab OR exp *"Limb"/ OR "limbs".ti,ab OR "limb".ti,ab OR "leg".ti,ab OR "legs".ti,ab OR "extremity".ti,ab OR "extremities".ti,ab OR "arm".ti,ab OR "arms".ti,ab OR "foot".ti,ab OR "feet".ti,ab OR "toe".ti,ab OR "toes".ti,ab OR "finger".ti,ab OR "fingers".ti,ab))) AND (*"Ankle Brachial Index"/ OR "Ankle Brachial Indices".ti,ab OR "Ankle-Brachial Index".ti,ab OR "Ankle-Brachial Indices".ti,ab OR "anklebrachial index".ti,ab OR "anklebrachialindex".ti,ab OR "ankle brachial".ti,ab OR "anklebrachial".ti,ab OR "ABI".ti,ab OR "Toe Brachial Index".ti,ab OR "Toe Brachial Indices".ti,ab OR "Toe-Brachial Index".ti,ab OR "Toe-Brachial Indices".ti,ab OR "Toe Pressure".ti,ab OR "toe brachial".ti,ab OR "toebrachial".ti,ab OR "TBI".ti,ab OR "TP".ti,ab OR exp *"Oximetry"/ OR "Oximetry".ti,ab OR "Oximetries".ti,ab OR "Oximetr*".ti,ab OR "Pulse Oximetries".ti,ab OR "Pulse Oximetry".ti,ab OR "transcutaneous oxygen tension".ti,ab OR "transcutaneous oxygen".ti,ab OR *"Pulse"/ OR "Pulse*".ti,ab OR "pulsation".ti,ab OR "pulsations".ti,ab OR *"Transcutaneous Oxygen Monitoring"/ OR "Transcutaneous Blood Gas Monitoring".ti,ab OR "Transcutaneous Capnometry".ti,ab OR "PtcO2".ti,ab OR "TcPCO2".ti,ab OR "Doppler waveform".ti,ab OR "Doppler waveforms".ti,ab OR "Doppler wave form".ti,ab OR "Doppler wave forms".ti,ab OR *"pulsed Doppler echocardiography"/ OR "Pulsed Doppler".ti,ab OR "Doppler Pulsed".ti,ab OR "non-invasive".ti,ab OR "non-invasiv*".ti,ab OR "point-of-care test".ti,ab OR "point-of-care tests".ti,ab OR *"Ankle Pressure"/ OR "Ankle Pressure".ti,ab OR "Ankle Pressures".ti,ab OR *"Skin Temperature"/ OR "Skin Temperature".ti,ab OR "Skin Temperatures".ti,ab OR exp *"Blood Pressure measurement"/ OR "blood pressure measurement".ti,ab OR "blood pressure measurements".ti,ab) AND (exp *"Wound Healing"/ OR "Wound Healing".ti,ab OR "Wounds Healing".ti,ab OR *"Ulcer Healing"/ OR "Ulcer Healing".ti,ab OR "Ulcers Healing".ti,ab OR (("Wound" ADJ4 "Healing") OR ("Wound" ADJ4 "Heal") OR ("Wound" ADJ4 "Healed") OR ("Wound" ADJ4 "Heals") OR ("Wounds" ADJ4 "Healing") OR ("Wounds" ADJ4 "Heal") OR ("Wounds" ADJ4 "Healed") OR ("Wounds" ADJ4 "Heals") OR ("Ulcer" ADJ4 "Healing") OR ("Ulcer" ADJ4 "Heal") OR ("Ulcer" ADJ4 "Healed") OR ("Ulcer" ADJ4 "Heals") OR ("Ulcers" ADJ4 "Healing") OR ("Ulcers" ADJ4 "Heal") OR ("Ulcers" ADJ4 "Healed") OR ("Ulcers" ADJ4 "Heals")).ti,ab OR (("predictor".ti,ab OR "predictors".ti,ab OR "predictive factor".ti,ab OR "predictive factors".ti,ab OR "predicting".ti,ab OR "Forecasting"/) AND (exp *"Amputation"/ OR "Amputation".ti,ab)) OR "Wound Outcome".ti,ab OR "Wound Outcomes".ti,ab OR "Ulcer Outcome".ti,ab OR "Ulcer Outcomes".ti,ab OR (("Wound" ADJ4 "Outcome") OR ("Wound" ADJ4 "Outcomes") OR ("Wounds" ADJ4 "Outcome") OR ("Wounds" ADJ4 "Outcomes") OR ("Ulcer" ADJ4 "Outcome") OR ("Ulcer" ADJ4 "Outcomes") OR ("Ulcers" ADJ4 "Outcome") OR ("Ulcers" ADJ4 "Outcomes")).ti,ab) AND ("Sensitivity and Specificity"/ OR "Sensitivity".ti,ab OR "Specificity".ti,ab OR "Predictive Value".ti,ab OR "ROC Curve".ti,ab OR "Signal-To-Noise".ti,ab OR "limit of detection".ti,ab OR "Cohort Analysis"/ OR exp "Follow Up"/ OR exp "Longitudinal Study"/ OR exp "Prospective Study"/ OR exp "Retrospective Study"/ OR "Cohort".ti,ab OR	604	120

	"Cohorts".ti,ab OR "Follow-Up".ti,ab OR "Longitudinal".ti,ab OR "Prospective".ti,ab OR "Retrospective".ti,ab OR exp "Cross-Sectional Study"/ OR "Cross-Sectional".ti,ab OR "Observational Study"/ OR "Observational Study".ti,ab OR "effectiveness".ti,ab OR "efficacy".ti,ab)) **OR** ((*"Peripheral Occlusive Artery Disease"/ OR "Peripheral Arterial Disease".ti,ab OR "Peripheral Arterial Diseases".ti,ab OR "Peripheral Artery Disease".ti,ab OR "Peripheral Artery Diseases".ti,ab OR "Peripheral Arterial Disorder".ti,ab OR "Peripheral Arterial Disorders".ti,ab OR "Peripheral Artery Disorder".ti,ab OR ("PAD".ti,ab AND ("artery".ti,ab OR "arteries".ti,ab OR "arterial".ti,ab)) OR "peripheral arterial occlusive disease".ti,ab OR "peripheral arterial occlusive diseases".ti,ab OR "peripheral artery occlusive disease".ti,ab OR "peripheral artery occlusive diseases".ti,ab OR "Peripheral Vascular Disease"/ OR "Peripheral Vascular Disease".ti,ab OR "Peripheral Vascular Diseases".ti,ab OR "Peripheral Vascular Disorder".ti,ab OR "Peripheral Vascular Disorders".ti,ab OR "Peripheral Angiopathies".ti,ab OR "Peripheral Angiopathy".ti,ab OR "Peripheral Arteriopathies".ti,ab OR "Peripheral Arteriopathy".ti,ab OR "Peripheral Vasculopathies".ti,ab OR "Peripheral Vasculopathy".ti,ab OR *"Monckeberg medial calcific sclerosis"/ OR "Monckeberg's Medial Calcific Sclerosis".ti,ab OR "Monckeberg's Sclerosis".ti,ab OR "Monckeberg Sclerosis".ti,ab OR ("Monckeberg*" ADJ3 "Sclerosis").ti,ab OR "Mönckeberg's Medial Calcific Sclerosis".ti,ab OR "Mönckeberg's Sclerosis".ti,ab OR "Mönckeberg Sclerosis".ti,ab OR ("Mönckeberg*" ADJ3 "Sclerosis").ti,ab OR "Monckeberg".ti,ab OR "Monckeberg*".ti,ab OR "Moenckeberg".ti,ab OR "Moenckeberg*".ti,ab OR "Medial Calcific Sclerosis".ti,ab OR ("Medial" ADJ3 "Calcific" ADJ3 "Scleroses").ti,ab OR "Mönckeberg Medial Calcific Sclerosis".ti,ab OR "incompressible arter*".ti,ab OR "incompressible vessel*".ti,ab OR ("medial" ADJ3 "calcified" ADJ3 "artery").ti,ab OR ("medial" ADJ3 "calcified" ADJ3 "arteries").ti,ab OR *"Intermittent Claudication"/ OR "Intermittent Claudication".ti,ab OR "Leriche Syndrome"/ OR "Leriche Syndrome".ti,ab OR "Leriche's Syndrome".ti,ab OR "Leriches Syndrome".ti,ab OR "Arteriosclerosis Obliterans"/ OR "Arteriosclerosis Obliterans".ti,ab OR ((exp *"Atherosclerosis"/ OR exp *"Arteriosclerosis"/ OR "Atherosclerosis".ti,ab OR "Arteriosclerosis".ti,ab) AND ("peripheral".ti,ab OR "peripheral*".ti,ab OR "limbs".ti,ab OR "limb".ti,ab OR "leg".ti,ab OR "legs".ti,ab OR "extremity".ti,ab OR "extremities".ti,ab OR "arm".ti,ab OR "arms".ti,ab)) OR "Fontaine IV".ti,ab OR *"Diabetic Foot"/ OR "Diabetic Foot".ti,ab OR "Diabetic Feet".ti,ab OR ((exp *"Diabetes Mellitus"/ OR "diabetes".ti,ab OR "diabet*".ti,ab OR exp *"Chronic Kidney Failure"/ OR "Chronic Kidney Disease".ti,ab OR "Chronic Kidney Diseases".ti,ab OR "Chronic Kidney Failure".ti,ab OR "Chronic Kidney Insufficiency".ti,ab OR "Chronic Renal Disease".ti,ab OR "Chronic Renal Diseases".ti,ab OR "Chronic Renal Failure".ti,ab OR "Chronic Renal Insufficiency".ti,ab OR "End Stage Kidney Disease".ti,ab OR "end stage renal disease"/ OR "End Stage Renal Disease".ti,ab OR "End-Stage Renal Failure".ti,ab OR "End-Stage Kidney Failure".ti,ab OR "ESRD".ti,ab) AND ("peripheral".ti,ab OR "peripheral*".ti,ab OR exp *"Limb"/ OR "limbs".ti,ab OR "limb".ti,ab OR "leg".ti,ab OR "legs".ti,ab OR "extremity".ti,ab OR "extremities".ti,ab OR "arm".ti,ab OR "arms".ti,ab OR "foot".ti,ab OR "feet".ti,ab OR "toe".ti,ab OR "toes".ti,ab OR "finger".ti,ab OR "fingers".ti,ab))) AND (*"Ankle Brachial Index"/ OR "Ankle Brachial Indices".ti,ab OR "Ankle-Brachial Index".ti,ab OR "Ankle-Brachial Indices".ti,ab OR "anklebrachial index".ti,ab OR "anklebrachialindex".ti,ab OR "ankle brachial".ti,ab OR "anklebrachial".ti,ab OR "ABI".ti,ab OR "Toe Brachial Index".ti,ab OR "Toe Brachial Indices".ti,ab OR "Toe-Brachial Index".ti,ab OR "Toe-Brachial Indices".ti,ab OR "Toe Pressure".ti,ab OR "toe brachial".ti,ab OR "toebrachial".ti,ab OR "TBI".ti,ab OR "TP".ti,ab OR exp *"Oximetry"/ OR "Oximetry".ti,ab OR "Oximetries".ti,ab OR "Oximetr*".ti,ab OR "Pulse Oximetries".ti,ab OR "Pulse Oximetry".ti,ab OR "transcutaneous oxygen tension".ti,ab OR "transcutaneous oxygen".ti,ab OR *"Pulse"/ OR "Pulse*".ti,ab OR "pulsation".ti,ab OR "pulsations".ti,ab OR *"Transcutaneous Oxygen Monitoring"/ OR "Transcutaneous Blood Gas Monitoring".ti,ab OR "Transcutaneous Capnometry".ti,ab OR "PtcO2".ti,ab OR "TcPCO2".ti,ab OR "Doppler waveform".ti,ab OR "Doppler waveforms".ti,ab OR "Doppler wave form".ti,ab OR "Doppler wave forms".ti,ab OR *"pulsed Doppler echocardiography"/ OR "Pulsed Doppler".ti,ab OR "Doppler Pulsed".ti,ab OR "non-invasive".ti,ab OR "non-invasiv*".ti,ab OR "point-of-care test".ti,ab OR "point-of-care tests".ti,ab OR *"Ankle Pressure"/ OR "Ankle Pressure".ti,ab OR "Ankle Pressures".ti,ab OR *"Skin Temperature"/ OR "Skin Temperature".ti,ab OR "Skin Temperatures".ti,ab OR exp *"Blood Pressure measurement"/ OR "blood pressure measurement".ti,ab OR "blood pressure measurements".ti,ab) AND (exp *"Wound Healing"/ OR "Wound Healing".ti OR "Wounds Healing".ti OR *"Ulcer Healing"/ OR "Ulcer Healing".ti OR "Ulcers Healing".ti OR (("Wound" ADJ4 "Healing") OR ("Wound" ADJ4 "Heal") OR ("Wound" ADJ4 "Healed") OR ("Wound" ADJ4 "Heals") OR ("Wounds" ADJ4 "Healing") OR ("Wounds" ADJ4 "Heal") OR ("Wounds" ADJ4 "Healed") OR ("Wounds" ADJ4 "Heals") OR ("Ulcer" ADJ4 "Healing") OR ("Ulcer" ADJ4 "Heal") OR ("Ulcer" ADJ4 "Healed") OR ("Ulcer" ADJ4 "Heals") OR ("Ulcers" ADJ4 "Healing") OR ("Ulcers" ADJ4 "Heal") OR ("Ulcers" ADJ4 "Healed") OR ("Ulcers" ADJ4 "Heals")).ti OR (("predictor".ti OR "predictors".ti OR "predictive factor".ti OR "predictive factors".ti OR "predicting".ti OR *"Forecasting"/) AND (exp *"Amputation"/ OR "Amputation".ti)) OR "Wound Outcome".ti OR "Wound Outcomes".ti OR "Ulcer Outcome".ti OR "Ulcer Outcomes".ti OR (("Wound" ADJ4 "Outcome") OR ("Wound" ADJ4 "Outcomes") OR ("Wounds" ADJ4 "Outcome") OR ("Wounds" ADJ4 "Outcomes") OR ("Ulcer" ADJ4 "Outcome") OR ("Ulcer" ADJ4 "Outcomes") OR ("Ulcers" ADJ4 "Outcome") OR ("Ulcers" ADJ4 "Outcomes")).ti))**)**		
Web of Science	**(**((TI=("Peripheral Occlusive Artery Disease" OR "Peripheral Arterial Disease" OR "Peripheral Arterial Diseases" OR "Peripheral Artery Disease" OR "Peripheral Artery Diseases" **OR "Peripheral Arterial Disorder" OR "Peripheral Arterial Disorders" OR "Peripheral Artery Disorder"** OR ("PAD" **AND ("artery" OR "arteries" OR "arterial"))** OR "peripheral arterial occlusive disease" OR "peripheral arterial occlusive diseases" OR "peripheral artery occlusive disease" OR "peripheral artery occlusive	423	37

	diseases" OR "Peripheral Vascular Disease" **OR "Peripheral Vascular Disease" OR "Peripheral Vascular Diseases" OR "Peripheral Vascular Disorder" OR "Peripheral Vascular Disorders"** OR "Peripheral Angiopathies" OR "Peripheral Angiopathy" OR "Peripheral Arteriopathies" OR "Peripheral Arteriopathy" OR "Peripheral Vasculopathies" OR "Peripheral Vasculopathy" OR "Monckeberg medial calcific sclerosis" OR "Monckeberg's Medial Calcific Sclerosis" OR "Monckeberg's Sclerosis" OR "Monckeberg Sclerosis" OR ("Monckeberg*" NEAR/3 "Sclerosis") OR "Mönckeberg's Medial Calcific Sclerosis" OR "Mönckeberg's Sclerosis" OR "Mönckeberg Sclerosis" OR ("Mönckeberg*" NEAR/3 "Sclerosis") OR "Monckeberg" OR "Monckeberg*" OR "Moenckeberg" OR "Moenckeberg*" OR "Medial Calcific Sclerosis" OR ("Medial" NEAR/3 "Calcific" NEAR/3 "Scleroses") OR "Mönckeberg Medial Calcific Sclerosis" OR "incompressible arter*" OR "incompressible vessel*" OR ("medial" NEAR/3 "calcified" NEAR/3 "artery") OR ("medial" NEAR/3 "calcified" NEAR/3 "arteries") OR "Intermittent Claudication" OR "Intermittent Claudication" OR "Leriche Syndrome" OR "Leriche Syndrome" OR "Leriche's Syndrome" OR "Leriches Syndrome" OR "Arteriosclerosis Obliterans" OR "Arteriosclerosis Obliterans" OR (("Atherosclerosis" OR "Arteriosclerosis" OR "Atherosclerosis" OR "Arteriosclerosis") AND ("peripheral" OR "peripheral*" OR "limbs" OR "limb" OR "leg" OR "legs" OR "extremity" OR "extremities" OR "arm" OR "arms")) **OR "Fontaine IV" OR "Diabetic Foot" OR "Diabetic Foot" OR "Diabetic Feet"** OR (("Diabetes Mellitus" OR "diabetes" OR "diabet*" OR "Chronic Kidney Failure" OR "Chronic Kidney Disease" OR "Chronic Kidney Diseases" OR "Chronic Kidney Failure" OR "Chronic Kidney Insufficiency" OR "Chronic Renal Disease" OR "Chronic Renal Diseases" OR "Chronic Renal Failure" OR "Chronic Renal Insufficiency" OR "End Stage Kidney Disease" OR "end stage renal disease" OR "End Stage Renal Disease" OR "End-Stage Renal Failure" OR "End-Stage Kidney Failure" OR "ESRD") AND ("peripheral" OR "peripheral*" **OR "Limb"** OR "limbs" OR "limb" OR "leg" OR "legs" OR "extremity" OR "extremities" OR "arm" OR "arms" **OR "foot" OR "feet" OR "toe" OR "toes" OR "finger" OR "fingers"**))) OR AK=("Peripheral Occlusive Artery Disease" OR "Peripheral Arterial Disease" OR "Peripheral Arterial Diseases" OR "Peripheral Artery Disease" OR "Peripheral Artery Diseases" **OR "Peripheral Arterial Disorder" OR "Peripheral Arterial Disorders" OR "Peripheral Artery Disorder"** OR ("PAD" **AND ("artery" OR "arteries" OR "arterial"))** OR "peripheral arterial occlusive disease" OR "peripheral arterial occlusive diseases" OR "peripheral artery occlusive disease" OR "peripheral artery occlusive diseases" OR "Peripheral Vascular Disease" **OR "Peripheral Vascular Disease" OR "Peripheral Vascular Diseases" OR "Peripheral Vascular Disorder" OR "Peripheral Vascular Disorders"** OR "Peripheral Angiopathies" OR "Peripheral Angiopathy" OR "Peripheral Arteriopathies" OR "Peripheral Arteriopathy" OR "Peripheral Vasculopathies" OR "Peripheral Vasculopathy" OR "Monckeberg medial calcific sclerosis" OR "Monckeberg's Medial Calcific Sclerosis" OR "Monckeberg's Sclerosis" OR "Monckeberg Sclerosis" OR ("Monckeberg*" NEAR/3 "Sclerosis") OR "Mönckeberg's Medial Calcific Sclerosis" OR "Mönckeberg's Sclerosis" OR "Mönckeberg Sclerosis" OR ("Mönckeberg*" NEAR/3 "Sclerosis") OR "Monckeberg" OR "Monckeberg*" OR "Moenckeberg" OR "Moenckeberg*" OR "Medial Calcific Sclerosis" OR ("Medial" NEAR/3 "Calcific" NEAR/3 "Scleroses") OR "Mönckeberg Medial Calcific Sclerosis" OR "incompressible arter*" OR "incompressible vessel*" OR ("medial" NEAR/3 "calcified" NEAR/3 "artery") OR ("medial" NEAR/3 "calcified" NEAR/3 "arteries") OR "Intermittent Claudication" OR "Intermittent Claudication" OR "Leriche Syndrome" OR "Leriche Syndrome" OR "Leriche's Syndrome" OR "Leriches Syndrome" OR "Arteriosclerosis Obliterans" OR "Arteriosclerosis Obliterans" OR (("Atherosclerosis" OR "Arteriosclerosis" OR "Atherosclerosis" OR "Arteriosclerosis") AND ("peripheral" OR "peripheral*" OR "limbs" OR "limb" OR "leg" OR "legs" OR "extremity" OR "extremities" OR "arm" OR "arms")) **OR "Fontaine IV" OR "Diabetic Foot" OR "Diabetic Foot" OR "Diabetic Feet"** OR (("Diabetes Mellitus" OR "diabetes" OR "diabet*" OR "Chronic Kidney Failure" OR "Chronic Kidney Disease" OR "Chronic Kidney Diseases" OR "Chronic Kidney Failure" OR "Chronic Kidney Insufficiency" OR "Chronic Renal Disease" OR "Chronic Renal Diseases" OR "Chronic Renal Failure" OR "Chronic Renal Insufficiency" OR "End Stage Kidney Disease" OR "end stage renal disease" OR "End Stage Renal Disease" OR "End-Stage Renal Failure" OR "End-Stage Kidney Failure" OR "ESRD") AND ("peripheral" OR "peripheral*" **OR "Limb"** OR "limbs" OR "limb" OR "leg" OR "legs" OR "extremity" OR "extremities" OR "arm" OR "arms" **OR "foot" OR "feet" OR "toe" OR "toes" OR "finger" OR "fingers"**))) OR AB=("Peripheral Occlusive Artery Disease" OR "Peripheral Arterial Disease" OR "Peripheral Arterial Diseases" OR "Peripheral Artery Disease" OR "Peripheral Artery Diseases" **OR "Peripheral Arterial Disorder" OR "Peripheral Arterial Disorders" OR "Peripheral Artery Disorder"** OR ("PAD" **AND ("artery" OR "arteries" OR "arterial"))** OR "peripheral arterial occlusive disease" OR "peripheral arterial occlusive diseases" OR "peripheral artery occlusive disease" OR "peripheral artery occlusive diseases" OR "Peripheral Vascular Disease" **OR "Peripheral Vascular Disease" OR "Peripheral Vascular Diseases" OR "Peripheral Vascular Disorder" OR "Peripheral Vascular Disorders"** OR "Peripheral Angiopathies" OR "Peripheral Angiopathy" OR "Peripheral Arteriopathies" OR "Peripheral Arteriopathy" OR "Peripheral Vasculopathies" OR "Peripheral Vasculopathy" OR "Monckeberg medial calcific sclerosis" OR "Monckeberg's Medial Calcific Sclerosis" OR "Monckeberg's Sclerosis" OR "Monckeberg Sclerosis" OR ("Monckeberg*" NEAR/3 "Sclerosis") OR "Mönckeberg's Medial Calcific Sclerosis" OR "Mönckeberg's Sclerosis" OR "Mönckeberg Sclerosis" OR ("Mönckeberg*" NEAR/3 "Sclerosis") OR "Monckeberg" OR "Monckeberg*" OR "Moenckeberg" OR "Moenckeberg*" OR "Medial Calcific Sclerosis" OR ("Medial" NEAR/3 "Calcific" NEAR/3 "Scleroses") OR "Mönckeberg Medial Calcific Sclerosis" OR "incompressible arter*" OR "incompressible vessel*" OR ("medial" NEAR/3 "calcified" NEAR/3 "artery") OR ("medial" NEAR/3 "calcified" NEAR/3 "arteries") OR "Intermittent Claudication" OR "Intermittent Claudication" OR "Leriche Syndrome" OR "Leriche Syndrome" OR "Leriche's Syndrome" OR "Leriches Syndrome" OR "Arteriosclerosis Obliterans" OR "Arteriosclerosis Obliterans" OR (("Atherosclerosis" OR		

	"Arteriosclerosis" OR "Atherosclerosis" OR "Arteriosclerosis") AND ("peripheral" OR "peripheral*" OR "limbs" OR "limb" OR "leg" OR "legs" OR "extremity" OR "extremities" OR "arm" OR "arms")) **OR "Fontaine IV" OR "Diabetic Foot" OR "Diabetic Foot" OR "Diabetic Feet"** OR (("Diabetes Mellitus" OR "diabetes" OR "diabet*" OR "Chronic Kidney Failure" OR "Chronic Kidney Disease" OR "Chronic Kidney Diseases" OR "Chronic Kidney Failure" OR "Chronic Kidney Insufficiency" OR "Chronic Renal Disease" OR "Chronic Renal Diseases" OR "Chronic Renal Failure" OR "Chronic Renal Insufficiency" OR "End Stage Kidney Disease" OR "end stage renal disease" OR "End Stage Renal Disease" OR "End-Stage Renal Failure" OR "End-Stage Kidney Failure" OR "ESRD") AND ("peripheral" OR "peripheral*" **OR "Limb"** OR "limbs" OR "limb" OR "leg" OR "legs" OR "extremity" OR "extremities" OR "arm" OR "arms" **OR "foot" OR "feet" OR "toe" OR "toes" OR "finger" OR "fingers"**)))) AND (TI=("Ankle Brachial Index" OR "Ankle Brachial Indices" OR "Ankle-Brachial Index" OR "Ankle-Brachial Indices" OR "anklebrachial index" OR "anklebrachialindex" OR "ankle brachial" OR "anklebrachial" OR "ABI" OR "Toe Brachial Index" OR "Toe Brachial Indices" OR "Toe-Brachial Index" OR "Toe-Brachial Indices" OR "Toe Pressure" OR "toe brachial" OR "toebrachial" OR "TBI" OR "TP" OR "Oximetry" OR "Oximetry" OR "Oximetries" OR "Oximetr*" OR "Pulse Oximetries" OR "Pulse Oximetry" OR "transcutaneous oxygen tension" OR "transcutaneous oxygen" OR "Pulse" OR "Pulse*" OR "pulsation" OR "pulsations" OR "Transcutaneous Oxygen Monitoring" OR "Transcutaneous Blood Gas Monitoring" OR "Transcutaneous Capnometry" OR "PtcO2" OR "TcPCO2" OR "Doppler waveform" OR "Doppler waveforms" OR "Doppler wave form" OR "Doppler wave forms" OR "pulsed Doppler echocardiography" OR "Pulsed Doppler" OR "Doppler Pulsed" OR "non-invasive" OR "non-invasiv*" OR "point-of-care test" OR "point-of-care tests" **OR "Ankle Pressure" OR "Ankle Pressure" OR "Ankle Pressures" OR "Skin Temperature" OR "Skin Temperature" OR "Skin Temperatures" OR "Blood Pressure measurement" OR "blood pressure measurement" OR "blood pressure measurements"**) OR AK=("Ankle Brachial Index" OR "Ankle Brachial Indices" OR "Ankle-Brachial Index" OR "Ankle-Brachial Indices" OR "anklebrachial index" OR "anklebrachialindex" OR "ankle brachial" OR "anklebrachial" OR "ABI" OR "Toe Brachial Index" OR "Toe Brachial Indices" OR "Toe-Brachial Index" OR "Toe-Brachial Indices" OR "Toe Pressure" OR "toe brachial" OR "toebrachial" OR "TBI" OR "TP" OR "Oximetry" OR "Oximetry" OR "Oximetries" OR "Oximetr*" OR "Pulse Oximetries" OR "Pulse Oximetry" OR "transcutaneous oxygen tension" OR "transcutaneous oxygen" OR "Pulse" OR "Pulse*" OR "pulsation" OR "pulsations" OR "Transcutaneous Oxygen Monitoring" OR "Transcutaneous Blood Gas Monitoring" OR "Transcutaneous Capnometry" OR "PtcO2" OR "TcPCO2" OR "Doppler waveform" OR "Doppler waveforms" OR "Doppler wave form" OR "Doppler wave forms" OR "pulsed Doppler echocardiography" OR "Pulsed Doppler" OR "Doppler Pulsed" OR "non-invasive" OR "non-invasiv*" OR "point-of-care test" OR "point-of-care tests" **OR "Ankle Pressure" OR "Ankle Pressure" OR "Ankle Pressures" OR "Skin Temperature" OR "Skin Temperature" OR "Skin Temperatures" OR "Blood Pressure measurement" OR "blood pressure measurement" OR "blood pressure measurements"**) OR AB=("Ankle Brachial Index" OR "Ankle Brachial Indices" OR "Ankle-Brachial Index" OR "Ankle-Brachial Indices" OR "anklebrachial index" OR "anklebrachialindex" OR "ankle brachial" OR "anklebrachial" OR "ABI" OR "Toe Brachial Index" OR "Toe Brachial Indices" OR "Toe-Brachial Index" OR "Toe-Brachial Indices" OR "Toe Pressure" OR "toe brachial" OR "toebrachial" OR "TBI" OR "TP" OR "Oximetry" OR "Oximetry" OR "Oximetries" OR "Oximetr*" OR "Pulse Oximetries" OR "Pulse Oximetry" OR "transcutaneous oxygen tension" OR "transcutaneous oxygen" OR "Pulse" OR "Pulse*" OR "pulsation" OR "pulsations" OR "Transcutaneous Oxygen Monitoring" OR "Transcutaneous Blood Gas Monitoring" OR "Transcutaneous Capnometry" OR "PtcO2" OR "TcPCO2" OR "Doppler waveform" OR "Doppler waveforms" OR "Doppler wave form" OR "Doppler wave forms" OR "pulsed Doppler echocardiography" OR "Pulsed Doppler" OR "Doppler Pulsed" OR "non-invasive" OR "non-invasiv*" OR "point-of-care test" OR "point-of-care tests" **OR "Ankle Pressure" OR "Ankle Pressure" OR "Ankle Pressures" OR "Skin Temperature" OR "Skin Temperature" OR "Skin Temperatures" OR "Blood Pressure measurement" OR "blood pressure measurement" OR "blood pressure measurements"**)) AND (TI=("Wound Healing" OR "Wound Healing" OR "Wounds Healing" OR "Ulcer Healing" OR "Ulcer Healing" OR "Ulcers Healing" OR (("Wound" NEAR/4 "Healing") OR ("Wound" NEAR/4 "Heal") OR ("Wound" NEAR/4 "Healed") OR ("Wound" NEAR/4 "Heals") OR ("Wounds" NEAR/4 "Healing") OR ("Wounds" NEAR/4 "Heal") OR ("Wounds" NEAR/4 "Healed") OR ("Wounds" NEAR/4 "Heals") OR ("Ulcer" NEAR/4 "Healing") OR ("Ulcer" NEAR/4 "Heal") OR ("Ulcer" NEAR/4 "Healed") OR ("Ulcer" NEAR/4 "Heals") OR ("Ulcers" NEAR/4 "Healing") OR ("Ulcers" NEAR/4 "Heal") OR ("Ulcers" NEAR/4 "Healed") OR ("Ulcers" NEAR/4 "Heals")) OR (("predictor" OR "predictors" OR "predictive factor" OR "predictive factors" OR "predicting" OR "Forecasting") AND ("Amputation" OR "Amputation")) **OR "Wound Outcome" OR "Wound Outcomes" OR "Ulcer Outcome" OR "Ulcer Outcomes" OR (("Wound" NEAR/4 "Outcome") OR ("Wound" NEAR/4 "Outcomes") OR ("Wounds" NEAR/4 "Outcome") OR ("Wounds" NEAR/4 "Outcomes") OR ("Ulcer" NEAR/4 "Outcome") OR ("Ulcer" NEAR/4 "Outcomes") OR ("Ulcers" NEAR/4 "Outcome") OR ("Ulcers" NEAR/4 "Outcomes")))**) OR AK=("Wound Healing" OR "Wound Healing" OR "Wounds Healing" OR "Ulcer Healing" OR "Ulcer Healing" OR "Ulcers Healing" OR (("Wound" NEAR/4 "Healing") OR ("Wound" NEAR/4 "Heal") OR ("Wound" NEAR/4 "Healed") OR ("Wound" NEAR/4 "Heals") OR ("Wounds" NEAR/4 "Healing") OR ("Wounds" NEAR/4 "Heal") OR ("Wounds" NEAR/4 "Healed") OR ("Wounds" NEAR/4 "Heals") OR ("Ulcer" NEAR/4 "Healing") OR ("Ulcer" NEAR/4 "Heal") OR ("Ulcer" NEAR/4 "Healed") OR ("Ulcer" NEAR/4 "Heals") OR ("Ulcers" NEAR/4 "Healing") OR ("Ulcers" NEAR/4 "Heal") OR ("Ulcers" NEAR/4 "Healed") OR ("Ulcers" NEAR/4 "Heals")) OR (("predictor" OR "predictors" OR "predictive factor" OR "predictive factors" OR "predicting" OR "Forecasting") AND ("Amputation" OR "Amputation")) **OR "Wound Outcome" OR**		

	**"Wound Outcomes" OR "Ulcer Outcome" OR "Ulcer Outcomes" OR (("Wound" NEAR/4 "Outcome") OR ("Wound" NEAR/4 "Outcomes") OR ("Wounds" NEAR/4 "Outcome") OR ("Wounds" NEAR/4 "Outcomes") OR ("Ulcer" NEAR/4 "Outcome") OR ("Ulcer" NEAR/4 "Outcomes") OR ("Ulcers" NEAR/4 "Outcome") OR ("Ulcers" NEAR/4 "Outcomes")))** OR AB=("Wound Healing" OR "Wound Healing" OR "Wounds Healing" OR "Ulcer Healing" OR "Ulcer Healing" OR "Ulcers Healing" OR (("Wound" NEAR/4 "Healing") OR ("Wound" NEAR/4 "Heal") OR ("Wound" NEAR/4 "Healed") OR ("Wound" NEAR/4 "Heals") OR ("Wounds" NEAR/4 "Healing") OR ("Wounds" NEAR/4 "Heal") OR ("Wounds" NEAR/4 "Healed") OR ("Wounds" NEAR/4 "Heals") OR ("Ulcer" NEAR/4 "Healing") OR ("Ulcer" NEAR/4 "Heal") OR ("Ulcer" NEAR/4 "Healed") OR ("Ulcer" NEAR/4 "Heals") OR ("Ulcers" NEAR/4 "Healing") OR ("Ulcers" NEAR/4 "Heal") OR ("Ulcers" NEAR/4 "Healed") OR ("Ulcers" NEAR/4 "Heals")) OR (("predictor" OR "predictors" OR "predictive factor" OR "predictive factors" OR "predicting" OR "Forecasting") AND ("Amputation" OR "Amputation")) **OR "Wound Outcome" OR "Wound Outcomes" OR "Ulcer Outcome" OR "Ulcer Outcomes" OR (("Wound" NEAR/4 "Outcome") OR ("Wound" NEAR/4 "Outcomes") OR ("Wounds" NEAR/4 "Outcome") OR ("Wounds" NEAR/4 "Outcomes") OR ("Ulcer" NEAR/4 "Outcome") OR ("Ulcer" NEAR/4 "Outcomes") OR ("Ulcers" NEAR/4 "Outcome") OR ("Ulcers" NEAR/4 "Outcomes"))**)) AND (TI=("Sensitivity and Specificity" OR "Sensitivity" OR "Specificity" OR "Predictive Value" OR "ROC Curve" OR "Signal-To-Noise" OR "limit of detection" OR "Cohort Analysis" OR "Follow Up" OR "Longitudinal Study" OR "Prospective Study" OR "Retrospective Study" OR "Cohort" OR "Cohorts" OR "Follow-Up" OR "Longitudinal" OR "Prospective" OR "Retrospective" OR "Cross-Sectional Study" OR "Cross-Sectional" OR "Observational Study" OR "Observational Study" OR "effectiveness" OR "efficacy") OR AK=("Sensitivity and Specificity" OR "Sensitivity" OR "Specificity" OR "Predictive Value" OR "ROC Curve" OR "Signal-To-Noise" OR "limit of detection" OR "Cohort Analysis" OR "Follow Up" OR "Longitudinal Study" OR "Prospective Study" OR "Retrospective Study" OR "Cohort" OR "Cohorts" OR "Follow-Up" OR "Longitudinal" OR "Prospective" OR "Retrospective" OR "Cross-Sectional Study" OR "Cross-Sectional" OR "Observational Study" OR "Observational Study" OR "effectiveness" OR "efficacy") OR AB=("Sensitivity and Specificity" OR "Sensitivity" OR "Specificity" OR "Predictive Value" OR "ROC Curve" OR "Signal-To-Noise" OR "limit of detection" OR "Cohort Analysis" OR "Follow Up" OR "Longitudinal Study" OR "Prospective Study" OR "Retrospective Study" OR "Cohort" OR "Cohorts" OR "Follow-Up" OR "Longitudinal" OR "Prospective" OR "Retrospective" OR "Cross-Sectional Study" OR "Cross-Sectional" OR "Observational Study" OR "Observational Study" OR "effectiveness" OR "efficacy"))) **OR** ((TI=("Peripheral Occlusive Artery Disease" OR "Peripheral Arterial Disease" OR "Peripheral Arterial Diseases" OR "Peripheral Artery Disease" OR "Peripheral Artery Diseases" **OR "Peripheral Arterial Disorder" OR "Peripheral Arterial Disorders" OR "Peripheral Artery Disorder"** OR ("PAD" **AND ("artery" OR "arteries" OR "arterial"))** OR "peripheral arterial occlusive disease" OR "peripheral arterial occlusive diseases" OR "peripheral artery occlusive disease" OR "peripheral artery occlusive diseases" OR "Peripheral Vascular Disease" **OR "Peripheral Vascular Disease" OR "Peripheral Vascular Diseases" OR "Peripheral Vascular Disorder" OR "Peripheral Vascular Disorders"** OR "Peripheral Angiopathies" OR "Peripheral Angiopathy" OR "Peripheral Arteriopathies" OR "Peripheral Arteriopathy" OR "Peripheral Vasculopathies" OR "Peripheral Vasculopathy" OR "Monckeberg medial calcific sclerosis" OR "Monckeberg's Medial Calcific Sclerosis" OR "Monckeberg's Sclerosis" OR "Monckeberg Sclerosis" OR ("Monckeberg*" NEAR/3 "Sclerosis") OR "Mönckeberg's Medial Calcific Sclerosis" OR "Mönckeberg's Sclerosis" OR "Mönckeberg Sclerosis" OR ("Mönckeberg*" NEAR/3 "Sclerosis") OR "Monckeberg" OR "Monckeberg*" OR "Moenckeberg" OR "Moenckeberg*" OR "Medial Calcific Sclerosis" OR ("Medial" NEAR/3 "Calcific" NEAR/3 "Scleroses") OR "Mönckeberg Medial Calcific Sclerosis" OR "incompressible arter*" OR "incompressible vessel*" OR ("medial" NEAR/3 "calcified" NEAR/3 "artery") OR ("medial" NEAR/3 "calcified" NEAR/3 "arteries") OR "Intermittent Claudication" OR "Intermittent Claudication" OR "Leriche Syndrome" OR "Leriche Syndrome" OR "Leriche's Syndrome" OR "Leriches Syndrome" OR "Arteriosclerosis Obliterans" OR "Arteriosclerosis Obliterans" OR (("Atherosclerosis" OR "Arteriosclerosis" OR "Atherosclerosis" OR "Arteriosclerosis") AND ("peripheral" OR "peripheral*" OR "limbs" OR "limb" OR "leg" OR "legs" OR "extremity" OR "extremities" OR "arm" OR "arms")) **OR "Fontaine IV" OR "Diabetic Foot" OR "Diabetic Foot" OR "Diabetic Feet"** OR (("Diabetes Mellitus" OR "diabetes" OR "diabet*" OR "Chronic Kidney Failure" OR "Chronic Kidney Disease" OR "Chronic Kidney Diseases" OR "Chronic Kidney Failure" OR "Chronic Kidney Insufficiency" OR "Chronic Renal Disease" OR "Chronic Renal Diseases" OR "Chronic Renal Failure" OR "Chronic Renal Insufficiency" OR "End Stage Kidney Disease" OR "end stage renal disease" OR "End Stage Renal Disease" OR "End-Stage Renal Failure" OR "End-Stage Kidney Failure" OR "ESRD") AND ("peripheral" OR "peripheral*" **OR "Limb"** OR "limbs" OR "limb" OR "leg" OR "legs" OR "extremity" OR "extremities" OR "arm" OR "arms" **OR "foot" OR "feet" OR "toe" OR "toes" OR "finger" OR "fingers"**))) OR AK=("Peripheral Occlusive Artery Disease" OR "Peripheral Arterial Disease" OR "Peripheral Arterial Diseases" OR "Peripheral Artery Disease" OR "Peripheral Artery Diseases" **OR "Peripheral Arterial Disorder" OR "Peripheral Arterial Disorders" OR "Peripheral Artery Disorder"** OR ("PAD" **AND ("artery" OR "arteries" OR "arterial"))** OR "peripheral arterial occlusive disease" OR "peripheral arterial occlusive diseases" OR "peripheral artery occlusive disease" OR "peripheral artery occlusive diseases" OR "Peripheral Vascular Disease" **OR "Peripheral Vascular Disease" OR "Peripheral Vascular Diseases" OR "Peripheral Vascular Disorder" OR "Peripheral Vascular Disorders"** OR "Peripheral Angiopathies" OR "Peripheral Angiopathy" OR "Peripheral Arteriopathies" OR "Peripheral Arteriopathy" OR "Peripheral Vasculopathies" OR "Peripheral Vasculopathy" OR "Monckeberg medial calcific sclerosis" OR "Monckeberg's Medial Calcific Sclerosis"		

	OR "Monckeberg's Sclerosis" OR "Monckeberg Sclerosis" OR ("Monckeberg*" NEAR/3 "Sclerosis") OR "Mönckeberg's Medial Calcific Sclerosis" OR "Mönckeberg's Sclerosis" OR "Mönckeberg Sclerosis" OR ("Mönckeberg*" NEAR/3 "Sclerosis") OR "Monckeberg" OR "Monckeberg*" OR "Moenckeberg" OR "Moenckeberg*" OR "Medial Calcific Sclerosis" OR ("Medial" NEAR/3 "Calcific" NEAR/3 "Scleroses") OR "Mönckeberg Medial Calcific Sclerosis" OR "incompressible arter*" OR "incompressible vessel*" OR ("medial" NEAR/3 "calcified" NEAR/3 "artery") OR ("medial" NEAR/3 "calcified" NEAR/3 "arteries") OR "Intermittent Claudication" OR "Intermittent Claudication" OR "Leriche Syndrome" OR "Leriche Syndrome" OR "Leriche's Syndrome" OR "Leriches Syndrome" OR "Arteriosclerosis Obliterans" OR "Arteriosclerosis Obliterans" OR (("Atherosclerosis" OR "Arteriosclerosis" OR "Atherosclerosis" OR "Arteriosclerosis") AND ("peripheral" OR "peripheral*" OR "limbs" OR "limb" OR "leg" OR "legs" OR "extremity" OR "extremities" OR "arm" OR "arms")) **OR "Fontaine IV" OR "Diabetic Foot" OR "Diabetic Foot" OR "Diabetic Feet"** OR (("Diabetes Mellitus" OR "diabetes" OR "diabet*" OR "Chronic Kidney Failure" OR "Chronic Kidney Disease" OR "Chronic Kidney Diseases" OR "Chronic Kidney Failure" OR "Chronic Kidney Insufficiency" OR "Chronic Renal Disease" OR "Chronic Renal Diseases" OR "Chronic Renal Failure" OR "Chronic Renal Insufficiency" OR "End Stage Kidney Disease" OR "end stage renal disease" OR "End Stage Renal Disease" OR "End-Stage Renal Failure" OR "End-Stage Kidney Failure" OR "ESRD") AND ("peripheral" OR "peripheral*" **OR "Limb"** OR "limbs" OR "limb" OR "leg" OR "legs" OR "extremity" OR "extremities" OR "arm" OR "arms" **OR "foot" OR "feet" OR "toe" OR "toes" OR "finger" OR "fingers"**))) OR AB=("Peripheral Occlusive Artery Disease" OR "Peripheral Arterial Disease" OR "Peripheral Arterial Diseases" OR "Peripheral Artery Disease" OR "Peripheral Artery Diseases" **OR "Peripheral Arterial Disorder" OR "Peripheral Arterial Disorders" OR "Peripheral Artery Disorder"** OR ("PAD" **AND ("artery" OR "arteries" OR "arterial"))** OR "peripheral arterial occlusive disease" OR "peripheral arterial occlusive diseases" OR "peripheral artery occlusive disease" OR "peripheral artery occlusive diseases" OR "Peripheral Vascular Disease" **OR "Peripheral Vascular Disease" OR "Peripheral Vascular Diseases" OR "Peripheral Vascular Disorder" OR "Peripheral Vascular Disorders"** OR "Peripheral Angiopathies" OR "Peripheral Angiopathy" OR "Peripheral Arteriopathies" OR "Peripheral Arteriopathy" OR "Peripheral Vasculopathies" OR "Peripheral Vasculopathy" OR "Monckeberg medial calcific sclerosis" OR "Monckeberg's Medial Calcific Sclerosis" OR "Monckeberg's Sclerosis" OR "Monckeberg Sclerosis" OR ("Monckeberg*" NEAR/3 "Sclerosis") OR "Mönckeberg's Medial Calcific Sclerosis" OR "Mönckeberg's Sclerosis" OR "Mönckeberg Sclerosis" OR ("Mönckeberg*" NEAR/3 "Sclerosis") OR "Monckeberg" OR "Monckeberg*" OR "Moenckeberg" OR "Moenckeberg*" OR "Medial Calcific Sclerosis" OR ("Medial" NEAR/3 "Calcific" NEAR/3 "Scleroses") OR "Mönckeberg Medial Calcific Sclerosis" OR "incompressible arter*" OR "incompressible vessel*" OR ("medial" NEAR/3 "calcified" NEAR/3 "artery") OR ("medial" NEAR/3 "calcified" NEAR/3 "arteries") OR "Intermittent Claudication" OR "Intermittent Claudication" OR "Leriche Syndrome" OR "Leriche Syndrome" OR "Leriche's Syndrome" OR "Leriches Syndrome" OR "Arteriosclerosis Obliterans" OR "Arteriosclerosis Obliterans" OR (("Atherosclerosis" OR "Arteriosclerosis" OR "Atherosclerosis" OR "Arteriosclerosis") AND ("peripheral" OR "peripheral*" OR "limbs" OR "limb" OR "leg" OR "legs" OR "extremity" OR "extremities" OR "arm" OR "arms")) **OR "Fontaine IV" OR "Diabetic Foot" OR "Diabetic Foot" OR "Diabetic Feet"** OR (("Diabetes Mellitus" OR "diabetes" OR "diabet*" OR "Chronic Kidney Failure" OR "Chronic Kidney Disease" OR "Chronic Kidney Diseases" OR "Chronic Kidney Failure" OR "Chronic Kidney Insufficiency" OR "Chronic Renal Disease" OR "Chronic Renal Diseases" OR "Chronic Renal Failure" OR "Chronic Renal Insufficiency" OR "End Stage Kidney Disease" OR "end stage renal disease" OR "End Stage Renal Disease" OR "End-Stage Renal Failure" OR "End-Stage Kidney Failure" OR "ESRD") AND ("peripheral" OR "peripheral*" **OR "Limb"** OR "limbs" OR "limb" OR "leg" OR "legs" OR "extremity" OR "extremities" OR "arm" OR "arms" **OR "foot" OR "feet" OR "toe" OR "toes" OR "finger" OR "fingers"**)))) AND (TI=("Ankle Brachial Index" OR "Ankle Brachial Indices" OR "Ankle-Brachial Index" OR "Ankle-Brachial Indices" OR "anklebrachial index" OR "anklebrachialindex" OR "ankle brachial" OR "anklebrachial" OR "ABI" OR "Toe Brachial Index" OR "Toe Brachial Indices" OR "Toe-Brachial Index" OR "Toe-Brachial Indices" OR "Toe Pressure" OR "toe brachial" OR "toebrachial" OR "TBI" OR "TP" OR "Oximetry" OR "Oximetry" OR "Oximetries" OR "Oximetr*" OR "Pulse Oximetries" OR "Pulse Oximetry" OR "transcutaneous oxygen tension" OR "transcutaneous oxygen" OR "Pulse" OR "Pulse*" OR "pulsation" OR "pulsations" OR "Transcutaneous Oxygen Monitoring" OR "Transcutaneous Blood Gas Monitoring" OR "Transcutaneous Capnometry" OR "PtcO2" OR "TcPCO2" OR "Doppler waveform" OR "Doppler waveforms" OR "Doppler wave form" OR "Doppler wave forms" OR "pulsed Doppler echocardiography" OR "Pulsed Doppler" OR "Doppler Pulsed" OR "non-invasive" OR "non-invasiv*" OR "point-of-care test" OR "point-of-care tests" **OR "Ankle Pressure" OR "Ankle Pressure" OR "Ankle Pressures" OR "Skin Temperature" OR "Skin Temperature" OR "Skin Temperatures" OR "Blood Pressure measurement" OR "blood pressure measurement" OR "blood pressure measurements"**) OR AK=("Ankle Brachial Index" OR "Ankle Brachial Indices" OR "Ankle-Brachial Index" OR "Ankle-Brachial Indices" OR "anklebrachial index" OR "anklebrachialindex" OR "ankle brachial" OR "anklebrachial" OR "ABI" OR "Toe Brachial Index" OR "Toe Brachial Indices" OR "Toe-Brachial Index" OR "Toe-Brachial Indices" OR "Toe Pressure" OR "toe brachial" OR "toebrachial" OR "TBI" OR "TP" OR "Oximetry" OR "Oximetry" OR "Oximetries" OR "Oximetr*" OR "Pulse Oximetries" OR "Pulse Oximetry" OR "transcutaneous oxygen tension" OR "transcutaneous oxygen" OR "Pulse" OR "Pulse*" OR "pulsation" OR "pulsations" OR "Transcutaneous Oxygen Monitoring" OR "Transcutaneous Blood Gas Monitoring" OR "Transcutaneous Capnometry" OR "PtcO2" OR "TcPCO2" OR "Doppler waveform" OR "Doppler waveforms" OR "Doppler wave form" OR "Doppler wave forms" OR "pulsed Doppler echocardiography" OR "Pulsed Doppler" OR "Doppler Pulsed" OR "non-invasive"		

	OR "non-invasiv*" OR "point-of-care test" OR "point-of-care tests" **OR "Ankle Pressure" OR "Ankle Pressure" OR "Ankle Pressures" OR "Skin Temperature" OR "Skin Temperature" OR "Skin Temperatures" OR "Blood Pressure measurement" OR "blood pressure measurement" OR "blood pressure measurements"**) OR AB=("Ankle Brachial Index" OR "Ankle Brachial Indices" OR "Ankle-Brachial Index" OR "Ankle-Brachial Indices" OR "anklebrachial index" OR "anklebrachialindex" OR "ankle brachial" OR "anklebrachial" OR "ABI" OR "Toe Brachial Index" OR "Toe Brachial Indices" OR "Toe-Brachial Index" OR "Toe-Brachial Indices" OR "Toe Pressure" OR "toe brachial" OR "toebrachial" OR "TBI" OR "TP" OR "Oximetry" OR "Oximetry" OR "Oximetries" OR "Oximetr*" OR "Pulse Oximetries" OR "Pulse Oximetry" OR "transcutaneous oxygen tension" OR "transcutaneous oxygen" OR "Pulse" OR "Pulse*" OR "pulsation" OR "pulsations" OR "Transcutaneous Oxygen Monitoring" OR "Transcutaneous Blood Gas Monitoring" OR "Transcutaneous Capnometry" OR "PtcO2" OR "TcPCO2" OR "Doppler waveform" OR "Doppler waveforms" OR "Doppler wave form" OR "Doppler wave forms" OR "pulsed Doppler echocardiography" OR "Pulsed Doppler" OR "Doppler Pulsed" OR "non-invasive" OR "non-invasiv*" OR "point-of-care test" OR "point-of-care tests" **OR "Ankle Pressure" OR "Ankle Pressure" OR "Ankle Pressures" OR "Skin Temperature" OR "Skin Temperature" OR "Skin Temperatures" OR "Blood Pressure measurement" OR "blood pressure measurement" OR "blood pressure measurements"**)) AND TI=("Wound Healing" OR "Wound Healing" OR "Wounds Healing" OR "Ulcer Healing" OR "Ulcer Healing" OR "Ulcers Healing" OR (("Wound" NEAR/4 "Healing") OR ("Wound" NEAR/4 "Heal") OR ("Wound" NEAR/4 "Healed") OR ("Wound" NEAR/4 "Heals") OR ("Wounds" NEAR/4 "Healing") OR ("Wounds" NEAR/4 "Heal") OR ("Wounds" NEAR/4 "Healed") OR ("Wounds" NEAR/4 "Heals") OR ("Ulcer" NEAR/4 "Healing") OR ("Ulcer" NEAR/4 "Heal") OR ("Ulcer" NEAR/4 "Healed") OR ("Ulcer" NEAR/4 "Heals") OR ("Ulcers" NEAR/4 "Healing") OR ("Ulcers" NEAR/4 "Heal") OR ("Ulcers" NEAR/4 "Healed") OR ("Ulcers" NEAR/4 "Heals")) OR (("predictor" OR "predictors" OR "predictive factor" OR "predictive factors" OR "predicting" OR "Forecasting") AND ("Amputation" OR "Amputation")) **OR "Wound Outcome" OR "Wound Outcomes" OR "Ulcer Outcome" OR "Ulcer Outcomes" OR (("Wound" NEAR/4 "Outcome") OR ("Wound" NEAR/4 "Outcomes") OR ("Wounds" NEAR/4 "Outcome") OR ("Wounds" NEAR/4 "Outcomes") OR ("Ulcer" NEAR/4 "Outcome") OR ("Ulcer" NEAR/4 "Outcomes") OR ("Ulcers" NEAR/4 "Outcome") OR ("Ulcers" NEAR/4 "Outcomes")))))**		
Cochrane Library	Three strings coupled to:<br><br>AND ("Sensitivity and Specificity" OR "Sensitivity" OR "Specificity" OR "Predictive Value" OR "ROC Curve" OR "Signal-To-Noise" OR "limit of detection" OR "Cohort Analysis" OR "Follow Up" OR "Longitudinal Study" OR "Prospective Study" OR "Retrospective Study" OR "Cohort" OR "Cohorts" OR "Follow-Up" OR "Longitudinal" OR "Prospective" OR "Retrospective" OR "Cross-Sectional Study" OR "Cross-Sectional" OR "Observational Study" OR "Observational Study" OR "effectiveness" OR "efficacy"):ti,ab,kw<br><br>(("Peripheral Occlusive Artery Disease" OR "Peripheral Arterial Disease" OR "Peripheral Arterial Diseases" OR "Peripheral Artery Disease" OR "Peripheral Artery Diseases" **OR "Peripheral Arterial Disorder" OR "Peripheral Arterial Disorders" OR "Peripheral Artery Disorder"** OR ("PAD" **AND ("artery" OR "arteries" OR "arterial"))** OR "peripheral arterial occlusive disease" OR "peripheral arterial occlusive diseases" OR "peripheral artery occlusive disease" OR "peripheral artery occlusive diseases" OR "Peripheral Vascular Disease" **OR "Peripheral Vascular Disease" OR "Peripheral Vascular Diseases" OR "Peripheral Vascular Disorder" OR "Peripheral Vascular Disorders"** OR "Peripheral Angiopathies" OR "Peripheral Angiopathy" OR "Peripheral Arteriopathies" OR "Peripheral Arteriopathy" OR "Peripheral Vasculopathies" OR "Peripheral Vasculopathy" OR "Monckeberg medial calcific sclerosis" OR "Monckeberg's Medial Calcific Sclerosis" OR "Monckeberg's Sclerosis" OR "Monckeberg Sclerosis" OR (Monckeberg* NEAR/3 Sclerosis) OR "Mönckeberg's Medial Calcific Sclerosis" OR "Mönckeberg's Sclerosis" OR "Mönckeberg Sclerosis" OR ("Mönckeberg*" NEAR/3 "Sclerosis") OR "Monckeberg" OR "Monckeberg*" OR "Moenckeberg" OR "Moenckeberg*" OR "Medial Calcific Sclerosis" OR ("Medial" NEAR/3 "Calcific" NEAR/3 "Scleroses") OR "Mönckeberg Medial Calcific Sclerosis" OR (incompressible NEXT arter*) OR (incompressible NEXT vessel*) OR ("medial" NEAR/3 "calcified" NEAR/3 "artery") OR ("medial" NEAR/3 "calcified" NEAR/3 "arteries") OR "Intermittent Claudication" OR "Intermittent Claudication" OR "Leriche Syndrome" OR "Leriche Syndrome" OR "Leriche's Syndrome" OR "Leriches Syndrome" OR "Arteriosclerosis Obliterans" OR "Arteriosclerosis Obliterans" OR (("Atherosclerosis" OR "Arteriosclerosis" OR "Atherosclerosis" OR "Arteriosclerosis") AND ("peripheral" OR "peripheral*" OR "limbs" OR "limb" OR "leg" OR "legs" OR "extremity" OR "extremities" OR "arm" OR "arms")) **OR "Fontaine IV" OR "Diabetic Foot" OR "Diabetic Foot" OR "Diabetic Feet"** OR (("Diabetes Mellitus" OR "diabetes" OR "diabet*" OR "Chronic Kidney Failure" OR "Chronic Kidney Disease" OR "Chronic Kidney Diseases" OR "Chronic Kidney Failure" OR "Chronic Kidney Insufficiency" OR "Chronic Renal Disease" OR "Chronic Renal Diseases" OR "Chronic Renal Failure" OR "Chronic Renal Insufficiency" OR "End Stage Kidney Disease" OR "end stage renal disease" OR "End Stage Renal Disease" OR "End-Stage Renal Failure" OR "End-Stage Kidney Failure" OR "ESRD") AND ("peripheral" OR "peripheral*" **OR "Limb"** OR "limbs" OR "limb" OR "leg" OR "legs" OR "extremity" OR "extremities" OR "arm" OR "arms" **OR "foot" OR "feet" OR "toe" OR "toes" OR "finger" OR "fingers"**))) AND ("Ankle Brachial Index" OR "Ankle Brachial	196	63

	Indices" OR "Ankle-Brachial Index" OR "Ankle-Brachial Indices" OR "anklebrachial index" OR "anklebrachialindex" OR "ankle brachial" OR "anklebrachial" OR "ABI" OR "Toe Brachial Index" OR "Toe Brachial Indices" OR "Toe-Brachial Index" OR "Toe-Brachial Indices" OR "Toe Pressure" OR "toe brachial" OR "toebrachial" OR "TBI" OR "TP" OR "Oximetry" OR "Oximetry" OR "Oximetries" OR "Oximetr*" OR "Pulse Oximetries" OR "Pulse Oximetry" OR "transcutaneous oxygen tension" OR "transcutaneous oxygen" OR "Pulse" OR "Pulse*" OR "pulsation" OR "pulsations" OR "Transcutaneous Oxygen Monitoring" OR "Transcutaneous Blood Gas Monitoring" OR "Transcutaneous Capnometry" OR "PtcO2" OR "TcPCO2" OR "Doppler waveform" OR "Doppler waveforms" OR "Doppler wave form" OR "Doppler wave forms" OR "pulsed Doppler echocardiography" OR "Pulsed Doppler" OR "Doppler Pulsed" OR "non-invasive" OR (non NEXT invasiv*) OR "point-of-care test" OR "point-of-care tests" **OR "Ankle Pressure" OR "Ankle Pressure" OR "Ankle Pressures" OR "Skin Temperature" OR "Skin Temperature" OR "Skin Temperatures" OR "Blood Pressure measurement" OR "blood pressure measurement" OR "blood pressure measurements"**) AND ("Wound Healing" OR "Wound Healing" OR "Wounds Healing" OR "Ulcer Healing" OR "Ulcer Healing" OR "Ulcers Healing" OR (("Wound" NEAR/4 "Healing") OR ("Wound" NEAR/4 "Heal") OR ("Wound" NEAR/4 "Healed") OR ("Wound" NEAR/4 "Heals") OR ("Wounds" NEAR/4 "Healing") OR ("Wounds" NEAR/4 "Heal") OR ("Wounds" NEAR/4 "Healed") OR ("Wounds" NEAR/4 "Heals") OR ("Ulcer" NEAR/4 "Healing") OR ("Ulcer" NEAR/4 "Heal") OR ("Ulcer" NEAR/4 "Healed") OR ("Ulcer" NEAR/4 "Heals") OR ("Ulcers" NEAR/4 "Healing") OR ("Ulcers" NEAR/4 "Heal") OR ("Ulcers" NEAR/4 "Healed") OR ("Ulcers" NEAR/4 "Heals")) OR (("predictor" OR "predictors" OR "predictive factor" OR "predictive factors" OR "predicting" OR "Forecasting") AND ("Amputation" OR "Amputation")) **OR "Wound Outcome" OR "Wound Outcomes"** OR **"Ulcer Outcome" OR "Ulcer Outcomes" OR (("Wound" NEAR/4 "Outcome") OR ("Wound" NEAR/4 "Outcomes") OR ("Wounds" NEAR/4 "Outcome") OR ("Wounds" NEAR/4 "Outcomes") OR ("Ulcer" NEAR/4 "Outcome") OR ("Ulcer" NEAR/4 "Outcomes") OR ("Ulcers" NEAR/4 "Outcome") OR ("Ulcers" NEAR/4 "Outcomes"))**)):ti,kw<br><br>(("Peripheral Occlusive Artery Disease" OR "Peripheral Arterial Disease" OR "Peripheral Arterial Diseases" OR "Peripheral Artery Disease" OR "Peripheral Artery Diseases" **OR "Peripheral Arterial Disorder" OR "Peripheral Arterial Disorders" OR "Peripheral Artery Disorder"** OR ("PAD" **AND ("artery" OR "arteries" OR "arterial"))** OR "peripheral arterial occlusive disease" OR "peripheral arterial occlusive diseases" OR "peripheral artery occlusive disease" OR "peripheral artery occlusive diseases" OR "Peripheral Vascular Disease" **OR "Peripheral Vascular Disease" OR "Peripheral Vascular Diseases" OR "Peripheral Vascular Disorder" OR "Peripheral Vascular Disorders"** OR "Peripheral Angiopathies" OR "Peripheral Angiopathy" OR "Peripheral Arteriopathies" OR "Peripheral Arteriopathy" OR "Peripheral Vasculopathies" OR "Peripheral Vasculopathy" OR "Monckeberg medial calcific sclerosis" OR "Monckeberg's Medial Calcific Sclerosis" OR "Monckeberg's Sclerosis" OR "Monckeberg Sclerosis" OR ("Monckeberg*" NEAR/3 "Sclerosis") OR "Mönckeberg's Medial Calcific Sclerosis" OR "Mönckeberg's Sclerosis" OR "Mönckeberg Sclerosis" OR ("Mönckeberg*" NEAR/3 "Sclerosis") OR "Monckeberg" OR "Monckeberg*" OR "Moenckeberg" OR "Moenckeberg*" OR "Medial Calcific Sclerosis" OR ("Medial" NEAR/3 "Calcific" NEAR/3 "Scleroses") OR "Mönckeberg Medial Calcific Sclerosis" OR "incompressible arter*" OR "incompressible vessel*" OR ("medial" NEAR/3 "calcified" NEAR/3 "artery") OR ("medial" NEAR/3 "calcified" NEAR/3 "arteries") OR "Intermittent Claudication" OR "Intermittent Claudication" OR "Leriche Syndrome" OR "Leriche Syndrome" OR "Leriche's Syndrome" OR "Leriches Syndrome" OR "Arteriosclerosis Obliterans" OR "Arteriosclerosis Obliterans" OR (("Atherosclerosis" OR "Arteriosclerosis" OR "Atherosclerosis" OR "Arteriosclerosis") AND ("peripheral" OR "peripheral*" OR "limbs" OR "limb" OR "leg" OR "legs" OR "extremity" OR "extremities" OR "arm" OR "arms")) **OR "Fontaine IV" OR "Diabetic Foot" OR "Diabetic Foot" OR "Diabetic Feet"** OR (("Diabetes Mellitus" OR "diabetes" OR "diabet*" OR "Chronic Kidney Failure" OR "Chronic Kidney Disease" OR "Chronic Kidney Diseases" OR "Chronic Kidney Failure" OR "Chronic Kidney Insufficiency" OR "Chronic Renal Disease" OR "Chronic Renal Diseases" OR "Chronic Renal Failure" OR "Chronic Renal Insufficiency" OR "End Stage Kidney Disease" OR "end stage renal disease" OR "End Stage Renal Disease" OR "End-Stage Renal Failure" OR "End-Stage Kidney Failure" OR "ESRD") AND ("peripheral" OR "peripheral*" **OR "Limb"** OR "limbs" OR "limb" OR "leg" OR "legs" OR "extremity" OR "extremities" OR "arm" OR "arms" **OR "foot" OR "feet" OR "toe" OR "toes" OR "finger" OR "fingers"**))):ti AND ("Ankle Brachial Index" OR "Ankle Brachial Indices" OR "Ankle-Brachial Index" OR "Ankle-Brachial Indices" OR "anklebrachial index" OR "anklebrachialindex" OR "ankle brachial" OR "anklebrachial" OR "ABI" OR "Toe Brachial Index" OR "Toe Brachial Indices" OR "Toe-Brachial Index" OR "Toe-Brachial Indices" OR "Toe Pressure" OR "toe brachial" OR "toebrachial" OR "TBI" OR "TP" OR "Oximetry" OR "Oximetry" OR "Oximetries" OR "Oximetr*" OR "Pulse Oximetries" OR "Pulse Oximetry" OR "transcutaneous oxygen tension" OR "transcutaneous oxygen" OR "Pulse" OR "Pulse*" OR "pulsation" OR "pulsations" OR "Transcutaneous Oxygen Monitoring" OR "Transcutaneous Blood Gas Monitoring" OR "Transcutaneous Capnometry" OR "PtcO2" OR "TcPCO2" OR "Doppler waveform" OR "Doppler waveforms" OR "Doppler wave form" OR "Doppler wave forms" OR "pulsed Doppler echocardiography" OR "Pulsed Doppler" OR "Doppler Pulsed" OR "non-invasive" OR "non-invasiv*" OR "point-of-care test" OR "point-of-care tests" **OR "Ankle Pressure" OR "Ankle Pressure" OR "Ankle Pressures" OR "Skin Temperature" OR "Skin Temperature" OR "Skin Temperatures" OR "Blood Pressure measurement" OR "blood pressure measurement" OR "blood pressure measurements"**):ti,ab,kw AND ("Wound Healing" OR "Wound Healing" OR "Wounds Healing" OR "Ulcer Healing" OR "Ulcer Healing" OR "Ulcers Healing" OR (("Wound" NEAR/4 "Healing") OR ("Wound" NEAR/4 "Heal") OR ("Wound" NEAR/4 "Healed") OR ("Wound" NEAR/4		

	"Heals") OR ("Wounds" NEAR/4 "Healing") OR ("Wounds" NEAR/4 "Heal") OR ("Wounds" NEAR/4 "Healed") OR ("Wounds" NEAR/4 "Heals") OR ("Ulcer" NEAR/4 "Healing") OR ("Ulcer" NEAR/4 "Heal") OR ("Ulcer" NEAR/4 "Healed") OR ("Ulcer" NEAR/4 "Heals") OR ("Ulcers" NEAR/4 "Healing") OR ("Ulcers" NEAR/4 "Heal") OR ("Ulcers" NEAR/4 "Healed") OR ("Ulcers" NEAR/4 "Heals")) OR (("predictor" OR "predictors" OR "predictive factor" OR "predictive factors" OR "predicting" OR "Forecasting") AND ("Amputation" OR "Amputation")) **OR "Wound Outcome" OR "Wound Outcomes" OR "Ulcer Outcome" OR "Ulcer Outcomes" OR (("Wound" NEAR/4 "Outcome") OR ("Wound" NEAR/4 "Outcomes") OR ("Wounds" NEAR/4 "Outcome") OR ("Wounds" NEAR/4 "Outcomes") OR ("Ulcer" NEAR/4 "Outcome") OR ("Ulcer" NEAR/4 "Outcomes") OR ("Ulcers" NEAR/4 "Outcome") OR ("Ulcers" NEAR/4 "Outcomes"))**)):ti,ab,kw)<br><br>(("Peripheral Occlusive Artery Disease" OR "Peripheral Arterial Disease" OR "Peripheral Arterial Diseases" OR "Peripheral Artery Disease" OR "Peripheral Artery Diseases" **OR "Peripheral Arterial Disorder" OR "Peripheral Arterial Disorders" OR "Peripheral Artery Disorder"** OR ("PAD" **AND ("artery" OR "arteries" OR "arterial"))** OR "peripheral arterial occlusive disease" OR "peripheral arterial occlusive diseases" OR "peripheral artery occlusive disease" OR "peripheral artery occlusive diseases" OR "Peripheral Vascular Disease" **OR "Peripheral Vascular Disease" OR "Peripheral Vascular Diseases" OR "Peripheral Vascular Disorder" OR "Peripheral Vascular Disorders"** OR "Peripheral Angiopathies" OR "Peripheral Angiopathy" OR "Peripheral Arteriopathies" OR "Peripheral Arteriopathy" OR "Peripheral Vasculopathies" OR "Peripheral Vasculopathy" OR "Monckeberg medial calcific sclerosis" OR "Monckeberg's Medial Calcific Sclerosis" OR "Monckeberg's Sclerosis" OR "Monckeberg Sclerosis" OR ("Monckeberg*" NEAR/3 "Sclerosis") OR "Mönckeberg's Medial Calcific Sclerosis" OR "Mönckeberg's Sclerosis" OR "Mönckeberg Sclerosis" OR ("Mönckeberg*" NEAR/3 "Sclerosis") OR "Monckeberg" OR "Monckeberg*" OR "Moenckeberg" OR "Moenckeberg*" OR "Medial Calcific Sclerosis" OR ("Medial" NEAR/3 "Calcific" NEAR/3 "Scleroses") OR "Mönckeberg Medial Calcific Sclerosis" OR "incompressible arter*" OR "incompressible vessel*" OR ("medial" NEAR/3 "calcified" NEAR/3 "artery") OR ("medial" NEAR/3 "calcified" NEAR/3 "arteries") OR "Intermittent Claudication" OR "Intermittent Claudication" OR "Leriche Syndrome" OR "Leriche Syndrome" OR "Leriche's Syndrome" OR "Leriches Syndrome" OR "Arteriosclerosis Obliterans" OR "Arteriosclerosis Obliterans" OR (("Atherosclerosis" OR "Arteriosclerosis" OR "Atherosclerosis" OR "Arteriosclerosis") AND ("peripheral" OR "peripheral*" OR "limbs" OR "limb" OR "leg" OR "legs" OR "extremity" OR "extremities" OR "arm" OR "arms")) **OR "Fontaine IV" OR "Diabetic Foot" OR "Diabetic Foot" OR "Diabetic Feet"** OR (("Diabetes Mellitus" OR "diabetes" OR "diabet*" OR "Chronic Kidney Failure" OR "Chronic Kidney Disease" OR "Chronic Kidney Diseases" OR "Chronic Kidney Failure" OR "Chronic Kidney Insufficiency" OR "Chronic Renal Disease" OR "Chronic Renal Diseases" OR "Chronic Renal Failure" OR "Chronic Renal Insufficiency" OR "End Stage Kidney Disease" OR "end stage renal disease" OR "End Stage Renal Disease" OR "End-Stage Renal Failure" OR "End-Stage Kidney Failure" OR "ESRD") AND ("peripheral" OR "peripheral*" **OR "Limb"** OR "limbs" OR "limb" OR "leg" OR "legs" OR "extremity" OR "extremities" OR "arm" OR "arms" **OR "foot" OR "feet" OR "toe" OR "toes" OR "finger" OR "fingers"**))):ti,kw AND ("Ankle Brachial Index" OR "Ankle Brachial Indices" OR "Ankle-Brachial Index" OR "Ankle-Brachial Indices" OR "anklebrachial index" OR "anklebrachialindex" OR "ankle brachial" OR "anklebrachial" OR "ABI" OR "Toe Brachial Index" OR "Toe Brachial Indices" OR "Toe-Brachial Index" OR "Toe-Brachial Indices" OR "Toe Pressure" OR "toe brachial" OR "toebrachial" OR "TBI" OR "TP" OR "Oximetry" OR "Oximetry" OR "Oximetries" OR "Oximetr*" OR "Pulse Oximetries" OR "Pulse Oximetry" OR "transcutaneous oxygen tension" OR "transcutaneous oxygen" OR "Pulse" OR "Pulse*" OR "pulsation" OR "pulsations" OR "Transcutaneous Oxygen Monitoring" OR "Transcutaneous Blood Gas Monitoring" OR "Transcutaneous Capnometry" OR "PtcO2" OR "TcPCO2" OR "Doppler waveform" OR "Doppler waveforms" OR "Doppler wave form" OR "Doppler wave forms" OR "pulsed Doppler echocardiography" OR "Pulsed Doppler" OR "Doppler Pulsed" OR "non-invasive" OR "non-invasiv*" OR "point-of-care test" OR "point-of-care tests" **OR "Ankle Pressure" OR "Ankle Pressure" OR "Ankle Pressures" OR "Skin Temperature" OR "Skin Temperature" OR "Skin Temperatures" OR "Blood Pressure measurement" OR "blood pressure measurement" OR "blood pressure measurements"**):ti AND ("Wound Healing" OR "Wound Healing" OR "Wounds Healing" OR "Ulcer Healing" OR "Ulcer Healing" OR "Ulcers Healing" OR (("Wound" NEAR/4 "Healing") OR ("Wound" NEAR/4 "Heal") OR ("Wound" NEAR/4 "Healed") OR ("Wound" NEAR/4 "Heals") OR ("Wounds" NEAR/4 "Healing") OR ("Wounds" NEAR/4 "Heal") OR ("Wounds" NEAR/4 "Healed") OR ("Wounds" NEAR/4 "Heals") OR ("Ulcer" NEAR/4 "Healing") OR ("Ulcer" NEAR/4 "Heal") OR ("Ulcer" NEAR/4 "Healed") OR ("Ulcer" NEAR/4 "Heals") OR ("Ulcers" NEAR/4 "Healing") OR ("Ulcers" NEAR/4 "Heal") OR ("Ulcers" NEAR/4 "Healed") OR ("Ulcers" NEAR/4 "Heals")) OR (("predictor" OR "predictors" OR "predictive factor" OR "predictive factors" OR "predicting" OR "Forecasting") AND ("Amputation" OR "Amputation")) **OR "Wound Outcome" OR "Wound Outcomes" OR "Ulcer Outcome" OR "Ulcer Outcomes" OR (("Wound" NEAR/4 "Outcome") OR ("Wound" NEAR/4 "Outcomes") OR ("Wounds" NEAR/4 "Outcome") OR ("Wounds" NEAR/4 "Outcomes") OR ("Ulcer" NEAR/4 "Outcome") OR ("Ulcer" NEAR/4 "Outcomes") OR ("Ulcers" NEAR/4 "Outcome") OR ("Ulcers" NEAR/4 "Outcomes"))**)):ti)		
Emcare	See Embase	183	11

Total			942
