# Supplementary material for: Prognostic performance of bedside tests for predicting ulcer healing and wound healing after minor amputation in patients prone to medial arterial calcification: A systematic review
Source: Vasc Med. 2025 Jan 21;30(2):250–60. doi: 10.1177/1358863X241309326 (PMC12014953; doi:10.1177/1358863X241309326)
Supplement: sj-pdf-2-vmj-10.1177_1358863X241309326 – Supplemental material for Prognostic performance of bedside tests for predicting ulcer healing and wound healing after minor amputation in patients prone to medial arterial calcification: A systematic review [file sj-pdf-2-vmj-10.1177_1358863X241309326.pdf]

TABLE S2: Overview of included studies regarding primary ulcer healing

Author & year (ref)	Country	Study design & setting	Population (n, age, gender, comorbidity)	Ulcer characteristics	Outcome definition	Point of care test	Prognostic performance		Comments
Apelqvist 1989[27]	Sweden	Prospective cohort study<br><br>Follow-up: every 6 months until healing was achieved.	N = 314 DFU patients<br><br>Mean age: 64 years<br>Male 49.6%<br>Mean DM duration: 17 years	Wagner grading system<br>Grade 1: n = 150 (47.8%)<br>Grade 2: n = 50 (15.9%)<br>Grade 3: n = 46 (14.6%)<br>Grade 4: n = 39 (12.4%)<br>Grade 5: n = 29 (9.2%)<br><br>Mean duration of ulcers at enrollment: 14.5 weeks.	Wound healing is defined as intact skin for at least 6 months or, for cases when the patient died within that period, as intact skin at the time of death.<br><br>Patients with several concurrent lesions are represented by the lesion with the worst outcome.	AP ≥ 40 mmHg<br><br>AP ≥ 80 mmHg<br><br>TP > 15 mmHg<br><br>TP > 45 mmHg	AP (40)<br>Sens: 100%<br>Spec: 11.6%<br>PLR: 1.1<br>NLR: 0.0<br><br>TP (15)<br>Sens: 96.1%<br>Spec: 32.6%<br>PLR: 1.4<br>NLR: 0.1	AP (80)<br>Sens: 87.1%<br>Spec: 42.9%<br>PLR: 1.5<br>NLR: 0.3<br><br>TP (45)<br>Sens: 77.4%<br>Spec: 75.0%<br>PLR: 3.1<br>NLR: 0.3	Patients were analysed according to their original AP/TP, also after revascularisation procedures.<br><br>For purposes of calculating predictive capabilities, we reclassified these patients to the group they belong in based on their new AP or TP.<br><br>Prognostic performance was calculated*.
Apelqvist 1990[28]	Sweden	Prospective cohort study<br><br>Follow-up: every 6 months until healing was achieved.	N = 314 DFU patients<br><br>Mean age: 64 years<br>Male: 49.7%<br>Mean DM duration: 17 years	Wagner grading system<br>Grade 1: 47.8%<br>Grade 2: 15.9%<br>Grade 3: 14.6%<br>Grade 4: 12.4%<br>Grade 5: 9.2%<br><br>Median duration ulceration 5 weeks (range 0 - 208)	Ulcer healing is defined as intact skin for at least 6 months or at time of death<br>Healing after amputation<br><br>Death before healing	Palpable femoral pulse<br><br>Palpable popliteal pulse<br><br>Palpable pedal pulse	Femoral<br>Sens: 98.5%<br>Spec: 3.4%<br>PLR: 1.0<br>NLR: 0.4<br><br>Pedal<br>Sens: 56.5%<br>Spec: 76.2%<br>PLR: 2.4<br>NLR: 0.6	Popliteal<br>Sens: 73.1%<br>Spec: 58.1%<br>PLR: 1.7<br>NLR: 0.5	Based on the size of the cohort and period of enrollment this study seems to concern the same cohort as Apelqvist et al. (1989). In this study 4 patients underwent revascularization procedures. This is not described in this article.

Ballard 1995[29]	United States	Prospective cohort study<br><br>Follow-up ranged from 2 to 15 months (mean 8 months). No loss to follow-up.	N = 55 patients, 66 feet<br><br>Mean age: 67 years<br>Male: 62%<br><br>N = 12 patients on dialysis (22%)	Treatment indications:<br>Non-healing ulcer 35 feet (53%)<br>Gangrene 25 feet (38%)<br>Rest pain 6 feet (9%)<br><br>Determination of treatment based on TcPO2 levels. ≥ 30mmHg conservative treatment. 30mmHg operative management with angioplasty or bypass surgery.	Treatment success or failure was defined as complete wound healing or relief of ischemic rest pain.	Palpable pedal pulses<br><br>ABI ≥ 0.60<br><br>TcPO2 ≥ 30mmHg	Pedal<br>Sens: 60%<br>Spec: 89%<br>PLR: 5.5<br>NLR: 0.45<br><br>$TcPO_2$<br>Sens: 98%<br>Spec: 44%<br>PLR: 1.8<br>NLR: 0.05	ABI<br>Sens: 94%<br>Spec: 40%<br>PLR: 1.6<br>NLR: 0.15	In the conservative group, 36 feet were monitored to end-point due to death 2 and 4 months after treatment initiation.<br><br>In the operative group, 28 limbs were monitored to end point because of primary lower limb amputation.<br><br>ABI could only be successfully measured in 41 of 62 eligible limbs due to incompressible vessels.
Bishara 2009[30]	Egypt	Prospective cohort study	N = 100 limbs, 62 patients<br><br>Mean age: 63 years<br>Male: 67.7%<br><br>43 limbs reached endpoint of healing.	Nonischemic lesions<br><br>Non-healing lesions<br><br>CLI (rest pain, ischemic ulcers/gangrene)	Healed wound (fully covered with intact skin)<br><br>Healing wound (completely covered with healthy granulations, with absence of tissue necrosis or infection)<br><br>Non-healing (no signs of granulation < 1 month or occurrence of CLI)	APSV ≥ 35 cm/s<br><br>APSV measured before and after revascularization.	APSV<br>Sens: 92.9%<br>Spec: 90.6%<br>PLR: 9.9<br>NLR: 0.02		If a patient presented with a foot lesion that did not heal and required revascularization, the patient was entered once initially as a non-healed lesion and entered once again after revascularization. If the inclusion criteria were applicable to both limbs in the same

									patient, each limb was entered separately.
Brechow 2013[37]	Germany	Prospective cohort study<br><br>6 monthly check up, total follow-up per patient of 2 years.	N = 678<br><br>Mean age: 66 years<br>Male: 69.3%<br>Mean DM duration: 30 years	Modified University of Texas Wound Classification System<br>1A n = 1 (0.1%)<br>1B n = 2 (0.3%)<br>1C n = 12 (1.8%)<br>1D n = 59 (8.7%)<br>2B n = 22 (3.2%)<br>2C n = 36 (5.3%<br>2D n = 218 (32.1%)<br>3B n = 16 (2.4%)<br>3C n = 9 (1.3%)<br>3D n = 257 (37.9%)<br>4B n = 2 (0.3%)<br>4C n= 4 (0.6%)<br>4D n = 60 (8.8%)<br><br>Overall<br>74% of ulcers healed at 2-year follow-up.	Complete healing without major amputation	ABI ≥ 0.5 (without signs of MAC)<br><br>ABI ≥ 0.9 (without signs of MAC)	ABI (0.5)<br>Sens: 49.3%<br>Spec: 56.3%<br>PLR: 1.1<br>NLR: 0.9	ABI (0.9)<br>Sens: 7.5%<br>Spec: 96.0%<br>PLR: 1.9<br>NLR: 1.0	It is unclear if major amputations occurred after the original wound had healed, or if all major amputations were performed because of primary healing failure.<br><br>It is unclear if ABI was re-examined after vascular interventions and if severity of PAOD was reclassified before analysis.<br><br>Prognostic performance was calculated based on estimated numbers in the figures of the article. Therefore, minor errors in our calculations could be present.*
Elghazaly 2023[31]	United Kingdom	Prospective observational comparison study<br><br>Follow-up: 12 months	N = 123 DFU<br><br>Mean age: 68 years<br>Gender: 77% male<br><br>Mean duration of DM = 18 years	According to Texas Grade:<br>- 0: 0.8%<br>- 1: 62.6%<br>- 2: 8.1%<br>- 3: 28.5%	Ulcer healing was defined as complete healing with full skin epithelialization (intact skin).<br><br>Healing of a wound after minor or major	ABI ≥ 0.9<br><br>AP ≥ 50 mmHg<br><br>TBI ≥ 0.8<br><br>TP ≥ 40 mmHg	ABI<br>Sens: 64%<br>Spec: 39%<br>PLR: 1.05<br>NLR: 0.92<br><br>TBI<br>Sens: 50%<br>Spec: 79%	AP<br>Sens: 7%<br>Spec: 86%<br>PLR: 0.50<br>NLR: 1.08<br><br>TP<br>Sens: 50%<br>Spec: 46%	Follow-up was performed prospectively for 12 months by regular quarterly review of electronic health records.

				Overall<br>52.8% of ulcers healed	amputation was not considered healing.	$TcPO_2 \geq 40$ mmHg<br><br>PAD-scan (monophasic, or absent)	PLR: 2.4<br>NLR: 0.63<br><br>$TcPO_2$<br>Sens: 70%<br>Spec: 43%<br>PLR: 1.4<br>NLR: 0.70	PLR: 0.9<br>NLR: 1.08<br><br>PAD-scan<br>Sens: 78%<br>Spec: 40%<br>PLR: 1.3<br>NLR: 0.56	Different thresholds for all point of care tests were analyzed; see reference for full elaboration.
Elgzyri 2013[47]	Sweden	Prospective observational study<br><br>Continuous follow-up until healing or death	N = 602 DFU<br><br>Mean age: 77 years<br>Gender: 60% male<br><br>Mean duration of DM = 15 years	At inclusion, 26% of patients had a deep ulcer (Wagner grade ≥3)<br><br>Overall<br>38% of ulcers healed	1) Primary healing (not specified)<br><br>2) Healed without major amputation	AP ≥ 50 mmHg<br><br>TP ≥ 30 mmHg	AP<br>Sens: 93%<br>Spec: 14%<br>PLR: 1.1<br>NLR: 0.48	TP<br>Sens: 55%<br>Spec: 52%<br>PLR: 1.1<br>NLR: 0.88	Patients were followed and treated according to a preset standardized protocol.<br><br>Healed without major amputation results can be obtained in the primary reference as well.
Elgzyri 2021[38]	Sweden	Prospective observational study (retrospectively analysed)<br><br>Follow-up: average of 41 weeks	N = 476 DFU<br><br>Mean age: 73 years<br>Gender: 63% male<br><br>63% of patients were insulin dependent	100% of ulcers had Wagner grade 4	Primary healing was defined as intact skin with complete epithelialization, also after auto or minor amputation	AP ≥ 80 mmHg<br><br>TP ≥ 30 mmHg	AP<br>Sens: 71%<br>Spec: 26%<br>PLR: 1.0<br>NLR: 1.09	TP<br>Sens: 44%<br>Spec: 34%<br>PLR: 0.67<br>NLR: 1.66	Healed auto or minor amputations were regarded as healing as well.<br><br>Ten patients were lost at follow-up.
Faris 1985[39]	Australia	Observational study<br><br>Follow-up time unclear	N = 61 DFU<br><br>Median age: 72 years<br>Gender: 61% male<br><br>Median duration of DM = 10 years	Wounds:<br>- 57% ulceration<br>- 43% gangrene	Ulcer healing included both primary healing and healing after local amputation	SPP > 40 mmHg	SPP<br>Sens: 97%<br>Spec: 80%<br>PLR: 4.9<br>NLR: 0.04		Unclear if design was prospective or retrospective.<br><br>Healed wounds after amputation were defined as healing as well, leading to an overestimation of

									prognostic performance.
Holstein 1980[40]	Denmark	Prospective cohort study<br><br>Follow-up: 45 months maximum	N = 35 DFU<br><br>Mean age: 68 years<br>Gender: 68% male<br><br>Mean duration of DM: 62.5% > 10 years<br>34% of patients were insulin dependent	An ulcer needed to be located on the toes or foot distal to the ankle joint and comprising a skin area of at least 5 by 5 mm and penetrating at least to the subcutis	Healing included minor amputation or resection of the foot as well (i.e. the limb was saved)	AP ≥ 50 mmHg<br><br>AP ≥ 80 mmHg<br><br>TP ≥ 30 mmHg<br><br>SPP ≥ 30 mmHg	AP (50)<br>Sens: 100%<br>Spec: 20%<br>PLR: 1.3<br>NLR: 0<br><br>TP<br>Sens: 72%<br>Spec: 100%<br>PLR: Inf<br>NLR: 0.28	AP (80)<br>Sens: 80%<br>Spec: 70%<br>PLR: 2.7<br>NLR: 0.28<br><br>SPP<br>Sens: 64%<br>Spec: 90%<br>PLR: 6.4<br>NLR: 0.40	Healed wounds after amputation were defined as healing as well, leading to an overestimation of prognostic performance.<br><br>Small sample size
Kalani 1999[44]	Sweden	Prospective observational study<br><br>Follow-up: 12 months	N = 50 DFU<br><br>Mean age: 61 years<br>Gender: 74% male<br><br>Mean duration of DM: 26 years<br>68% of patients were insulin dependent	Chronic foot ulcers of >2 months duration<br><br>Average size 230 mm^2	Healed with intact skin or improved ulcer healing	$TcPO_2$ ≥25 mmHg<br><br>TBP ≥ 30 mmHg and ≥ 45 mmHg	TBP (30)<br>Sens: 15%<br>Spec: 97%<br>PLR: 5.0<br>NLR: 0.88<br><br>TBP (45)<br>Sens: 46%<br>Spec: 84%<br>PLR: 2.9<br>NLR: 0.64	$TcPO_2$<br>Sens: 85%<br>Spec: 92%<br>PLR: 6.0<br>NLR: 0.09	Improved ulcer healing (change of 25% area reduction) after 12 months was still considered 'healed'.<br><br>Small sample size.
Karanfilian 1986[45]	United States of America	Retrospective observational study<br><br>Follow-up: maximum of 4 months	N = 37 diabetic patients<br><br>Mean age: 57 years<br>Gender: 100% male	Chronic foot ulcers, digital and transmetatarsal amputations were all included	Healing was defined as closed wound edges.<br><br>If after 30 days there was no evidence of healthy granulation tissue, wound contraction, or a viable graft, the ulcer was classified as nonhealing.	$TcPO_2$ > 10 mmHg<br><br>LDV > 40 mV<br><br>AP > 30 mmHg	$TcPO_2$<br>Sens: 100%<br>Spec: 83%<br>PLR: 6.0<br>NLR: 0.0<br><br>LDV<br>Sens: 88%<br>Spec: 94%<br>PLR: 14.9<br>NLR: 0.13	AP<br>Sens: 65%<br>Spec: 15%<br>PLR: 0.8<br>NLR: 2.35	Very small sample size<br><br>Chronic DFU and wounds after amputation were included in the same patient group

Kawai 2017[22]	China	Retrospective cohort study<br><br>Follow-up: maximum of 10 months	N = 117 DFU (65 patients)<br><br>Mean age: 70 years<br>Gender: NS	No information about ulcer severity	Definition of healing not specifically mentioned	SPP > 43 mmHg	SPP<br>Sens: 67%<br>Spec: 94%<br>PLR: 11.7<br>NLR: 0.35		Lack of information about patient inclusion and ulcer severity.
Ladurner 2010[32]	Germany	Prospective cohort study<br><br>Follow-up: 1 year	N = 141 DFU<br><br>Mean age: 72 years<br>Gender: 62% male	No information about ulcer severity	Healing was defined as complete epithelization	$TcPO_2$ > 20 mmHg	$TcPO_2$<br>Sens: 76%<br>Spec: 56%<br>PLR: 1.7<br>NLR: 0.43		All patients were ineligible for revascularization.<br><br>Healing after minor and major amputation is not shown here.
Lee 2019[48]	South-Korea	Retrospective observational study<br><br>Follow-up time unclear	N = 263 DFU<br><br>Mean age: 62 years<br>Gender: 75% male<br><br>At least diagnosed with DM for 5 years	Ulcers needed to be located at the forefoot area<br><br>According to Texas Grade:<br>- 1: 27%<br>- 2: 5%<br>- 3: 67%<br><br>Average ulcer size was 7.5 cm^2<br>Average duration of ulcer was 10 weeks	Definition of healing not specifically mentioned	SHL > poor<br><br>$TcPO_2$ > 40 mmHg	SHL<br>Sens: 62%<br>Spec: 54%<br>PLR: 1.3<br>NLR: 0.72	$TcPO_2$<br>Sens: 47%<br>Spec: 68%<br>PLR: 1.5<br>NLR: 0.78	Only results without revascularization are shown here.<br><br>Indication to perform major or minor amputation were regarded as non-healing.
Lee 2022[49]	South-Korea	Retrospective observational study<br><br>Follow-up time unclear	N = 834 DFU<br><br>Mean age: 62 years<br>Gender: 74% male<br><br>At least diagnosed with DM for 5 years	Ulcers needed to be located at the forefoot area<br><br>According to Texas Grade:<br>- 1: 31%<br>- 2: 6%<br>- 3: 63%	Definition of healing not specifically mentioned	SHL ≥ 21	SHL<br>Sens: 72%<br>Spec: 70%<br>PLR: 2.4<br>NLR: 0.40		

				Average ulcer size was 8.1 cm^2<br>Average duration of ulcer was 11 weeks					
Leenstra 2020[33]	The Netherlands	Prospective observational study<br><br>Follow-up: 12 months	N = 103 DFU<br><br>Mean age: 70 years<br>Gender: 70% male	Most ulcers were located at the toes<br><br>The majority of wounds were classified as W1I0/1FI0	Healing was defined as fully healed ulcers and non-healing as ulcers that deteriorated under conservative treatment or that required surgical amputation	$TcPO_2$ > 43 mmHg	$TcPO_2$<br>Sens: 78%<br>Spec: 56%<br>PLR: 1.8<br>NLR: 0.39		Patients were excluded if they underwent revascularization or had any clinical signs of sepsis.
López-Moral 2022[34]	Spain	Prospective cohort study<br><br>Follow-up: 12 months	N = 21 DFU<br><br>Mean age: 69 years<br>Gender: 81% male<br><br>Mean duration of DM: 21 years	Average ulcer severity: SINBAD 4±1.09<br><br>Average wound area was 2.38 cm^2	Healing was defined as complete epithelization without any drainage confirmed for at least 10 days after closure	ABI > 0.52<br><br>TBI > 0.65<br><br>$TcPO_2$ > 28.5 mmHg	ABI<br>Sens: 100%<br>Spec: 75%<br>PLR: 4.0<br>NLR: 0<br><br>$TcPO_2$<br>Sens: 91%<br>Spec: 100%<br>PLR: 0<br>NLR: 0.09	TBI<br>Sens: 72%<br>Spec: 100%<br>PLR: 0<br>NLR: 0.28	14% of patients had revascularization during study follow-up<br><br>Very small sample size
Manu 2021[41]	United Kingdom	Retrospective cohort study<br><br>Follow-up: 12 months	N = 128 DFU<br><br>Mean age: 62 years<br>Gender: 79% male<br><br>Mean duration of DM: 22 years	Most ulcers were located at the toes and sole<br><br>Median SINBAD score: 3<br><br>Median WIfI score: 3	Healing was deducted from clinical notes<br><br>Healed wounds after amputation were regarded healing as well	ABI ≥ 0.9<br><br>TBI ≥ 0.75	ABI (≥ 0.9)<br>Sens: 83%<br>Spec: 22%<br>PLR: 1.1<br>NLR: 0.78	TBI (≥ 0.75)<br>Sens: 26%<br>Spec: 71%<br>PLR: 0.9<br>NLR: 1.05	16% of patients were lost to follow-up / no complete data.
Mennes 2021[35]	The Netherlands	Prospective observational study	N = 53 DFU	70% of ulcers were UT-classifcation 0	Healing was defined as complete re-epithelialization and	ABI > 0.89<br><br>TBI > 0.51	ABI<br>Sens: 62%<br>Spec: 63%	TBI<br>Sens: 74%<br>Spec: 50%	Patients with revascularization procedures during

		Follow-up: 6 months	Mean age: 67 years<br>Gender: 79% male<br><br>Mean duration of DM: 57% > 10 years	or 1	scored at 12 and 26 weeks	TP > 54 mmHg<br><br>AP > 96 mmHg<br><br>$TcPO_2$ > 30.5 mmHg	PLR: 1.7<br>NLR: 0.61<br><br>AP<br>Sens: 82%<br>Spec: 44%<br>PLR: 1.5<br>NLR: 0.40<br><br>$TcPO_2$<br>Sens: 85%<br>Spec: 31%<br>PLR: 1.2<br>NLR: 0.47	PLR: 1.5<br>NLR: 0.53<br><br>TP<br>Sens: 82%<br>Spec: 44%<br>PLR: 1.5<br>NLR: 0.40	follow-up were excluded.<br><br>Patients with major amputation were excluded.
Nouvong 2009[36]	United States of America	Prospective observational study (blinded)<br><br>Follow-up: 6 months	N = 73 DFU<br><br>Mean age: 50 years<br>Gender: 88% male<br><br>Mean duration of DM: 13 years	Ulcer severity not specified	Healing was defined as complete re-epithelialization and no exudates	ABI > 0.90	ABI<br>Sens: 90%<br>Spec: 21%<br>PLR: 1.1<br>NLR: 0.48		The treating physicians were blinded to the data.<br><br>No criteria for wound size or duration were used to select patients.
Padberg 1996[23]	United States of America	Prospective observational study<br><br>Follow-up time unclear	N = 129 DFU in CLTI patients<br><br>N = 22 wounds in patients with chronic renal failure<br><br>Demographic data not presented	Ulcer severity not specified<br><br>DM<br>54% of ulcers healed<br><br>CRF<br>50% of ulcers healed	Healing was defined as complete wound closure, with epithelialization of the wound surface or a healed suture line	**DM**<br>ABI ~>0.5<br><br>$TcPO_2$ ~>20mmHg<br><br>**CRF**<br>ABI ~>1.1<br><br>$TcPO_2$ ~>25mmHg	**DM**<br>ABI<br>Sens: 81%<br>Spec: 31%<br>PLR: 1.2<br>NLR: 0.61<br><br>**CFR**<br>ABI<br>Sens: 46%<br>Spec: 55%<br>PLR: 1.0<br>NLR: 0.98	$TcPO_2$<br>Sens: 81%<br>Spec: 81%<br>PLR: 4.3<br>NLR: 0.23<br><br>$TcPO_2$<br>Sens: 73%<br>Spec: 82%<br>PLR: 4.1<br>NLR: 0.33	Cut-off values not clearly described. Only estimation on graph possible.<br><br>Heel ulcers, limbs with uncontrolled foot infections, or those with an obvious neuropathic etiology were excluded.
Rajagopalan 2018[42]	India	Prospective observational study	N = 564 DFU<br><br>Mean age: 58 years	Only foot ulcers with Wagner	Healing was defined as complete epithelialization of the	ABI ≥0.9	ABI<br>Sens: 90%<br>Spec: 42%	$TcPO_2$<br>Sens: 84%<br>Spec: 44%	Healing of amputation stump was regarded as

		Follow-up: 6 months	Gender: 67% male<br><br>Mean duration of DM: 12 years	Grade 2 or 3 were included<br><br>83% of ulcers healed with 42.6 mean healing days	ulcer or amputation stump	$TcPO_2$ >40 mmHg	PLR: 1.53<br>NLR: 0.25	PLR: 1.5<br>NLR: 0.36	primary healing as well.<br><br>5 patients who underwent revascularization procedures and 13 patients with a non-healing ulcer were excluded.<br><br>Specificity of 97% was reported for ABI <0.9, however this result could not be reproduced based on available data. A specificity of 90% was calculated based on data provided in the article.<br><br>Prevalence of PAD was low (18%).
Thottiyen 2023[46]	India	Longitudinal observational study<br><br>Follow-up: 3 months	N = 121 DFU<br><br>Mean age: 65 years<br>Gender: 74% male<br><br>Chronic Kidney Disease was present in 21% of patients	The ulcer should be below the level of the ankle joint and belong to Wagner's grades 1 to 4 without palpable pulses of the dorsalis pedis and posterior tibial artery.	Healing was defined as complete epithelialization or healing changes such as granulation tissue	ABI >0.65<br><br>$TcPO_2$ >27.5 mmHg	ABI<br>Sens: 86%<br>Spec: 76%<br>PLR: 3.6<br>NLR: 0.18	$TcPO_2$<br>Sens: 85%<br>Spec: 82%<br>PLR: 4.7<br>NLR: 0.18	A change in healing status was already defined as 'healing', possibly leading to an overestimation of diagnostic performance.<br><br>Short follow-up time.
Vincente Jiménez 2015[24]	Spain	Prospective cohort	N = 19 patients	University of Texas classification	Healing vs. non-healing	$TcPO_2 \geq 35$ mmHg (after revascularization)	$TcPO_2$<br>Sens: 90.0%<br>Spec: 88.9%		Small population, mean age not provided, also not

		Follow-up time unclear	Mean age: Not provided<br>Male: 72%	1c: 26.1%, n = 5<br>2c: 10.5%, n = 2<br>2d: 15.8%, n = 3<br>3c: 10.5%, n = 2<br>3d: 36.9%, n = 7<br><br>Healing rate: 53%<br>Median time to healing: 103 days		TcPO2 measured on dorsum of foot before and after revascularization	PLR: 8.1<br>NLR: 0.11		possible to calculate.<br><br>No clear definition of healing. Also not specified if healing after minor amputation was considered healing.
Wallin 1989[43]	Sweden	Prospective cohort<br><br>Follow-up time unclear	N = 83<br><br>Mean age: 70 years<br>Male: 55.4%<br><br>N = 68 diabetics	No wound classification provided	Major amputation: all amputations above the ankle<br><br>Healing: Healing without major amputation (either after conservative treatment or minor foot operation including toe/transmetatarsal amputations)	AP ≥ 70 mmHg<br><br>TP ≥ 20 mmHg	AP<br>Sens: 91.7%<br>Spec: 73.3%<br>PLR: 3.4<br>NLR: 0.1	TP<br>Sens: 87.8%<br>Spec: 78.6%<br>PLR: 4.1<br>NLR: 0.2	Both primary healing and healing after minor operation are considered healing.<br><br>Prognostic performance was calculated*.
Yang 2013[26]	China	Prospective cohort<br><br>Follow-up time: average of 12 months	N = 61<br><br>Mean age and gender not reported for entire cohort	Wagner grading system<br>Grade 2 n = 38 (62.2%)<br>Grade 3 n = 8 (13.1%)<br>Grade 4 n = 17 (27.9%)	Ulcers healed with intact skin<br><br>Improved ulcers (reduction wound size ≥ 50%)<br><br>Unimproved or worsened ulcers	$TcPO_2$ ≥ 25 mmHg	$TcPO_2$<br>Sens: 88.6%<br>Spec: 82.4%<br>PLR: 5.0<br>NLR: 0.14		

Abbreviations: ABI = ankle branchial index, AP = ankle pressure, APSV = ankle peak systolic velocity, CLI = Critical limb ischemia, DFU = diabetic foot ulcer, DM = diabetes mellitus, LDV = laser doppler velocimetry, PAD = podiatry ankle scan, PAOD = peripheral arterial occlusive disease, PLR = positive likelihood ratio, MAC = medial arterial calcification, NLR = negative likelihood ratio, sens = sensitivity, spec = specificity, SHL = skin hydration level, SPP = skin perfusion pressure, TBI = toe brachial index, TBP = toe blood pressure, TcPO2 = transcutaneous oxygen pressure, TP = toe pressure

*** =** Predictive capabilities were calculated based on numbers provided in the article. If no clear cut-off values were mentioned, we chose cut-off values based on
1) how clearly we could distinguish the necessary data from the figures or text and
2) the cut-off values commonly used in other articles.

TABLE S3: Overview of included studies regarding wound healing after minor amputation

Author & year (ref)	Country	Study design & setting	Population (n, age, gender, comorbidity)	Ulcer characteristics	Outcome definition	Point of care test	Prognostic performance		Comments
Gibbons 1979[50]	United States of America	Prospective observational study<br><br>Follow-up time unclear	N = 66 diabetic patients needing forefoot amputation<br><br>Mean age: 64 years<br>Gender: 67% male<br><br>Mean duration of DM: 19.5 years<br>55% of patients were insulin dependent	If clinical assessment indicated a reasonable chance of healing, amputation was performed	Healing of forefoot (toe, metatarsal head or transmetatarsal) amputation	AP ≥70 mmHg<br><br>PVR (flat or slight)	AP<br>Sens: 64%<br>Spec: 33%<br>PLR: 1.0<br>NLR: 1.09	PVR<br>Sens: 91%<br>Spec: 50%<br>PLR: 1.8<br>NLR: 0.18	No description of initial wounds.<br><br>No explanation on which patients were excluded.<br><br>In three patients in the healed amputation group, no forefoot pulse volume recording was obtained because of the proximity of the ulcer.
Holstein 1984[51]	Denmark	Retrospective cohort study<br><br>Follow-up: at least 7 years	N = 102 diabetic patients (109 diabetic feet)<br><br>Mean age: 65 years<br>Gender: 67% male	Out of the 109 diabetic feet, there were 102 with infection. In 40 feet, the infection was invasive in form of osteitis and/or plantar abscess.	Healing of digital and forefoot amputations<br><br>Definition of healing not specifically mentioned	SDBP ≥ 30 mmHg (TP)<br><br>SABP ≥50 mmHg (AP)<br><br>SPFF ≥ 40 mmHg	SDBP<br>Sens: 67%<br>Spec: 50%<br>PLR: 1.3<br>NLR: 0.66<br><br>SPFF<br>Sens: 56%<br>Spec: 54%<br>PLR: 1.2<br>NLR: 0.81	SABP<br>Sens: 100%<br>Spec: 9%<br>PLR: 1.1<br>NLR: 0.00	Subgroup analysis for DM was performed.<br><br>Statistical analysis not clearly explained.<br><br>Time and definition of healing was not mentioned.

Larsson 1993[52]	Sweden	Prospective observational study<br><br>Follow-up: 6 months	N = 159 DFU requiring amputation<br><br>Mean age: 70 years<br>Gender: 54% male<br><br>Mean duration of DM: 18 years	Ulcer severity: 89% had Wagner grade ≥3	Healing was defined as intact skin (after minor amputation)	ABI ≥0.5<br><br>AP ≥ 75 mmHg<br><br>TP ≥ 15 mmHg<br><br>TBI ≥ 0.10	<u>ABI</u><br>Sens: 94%<br>Spec: 53%<br>PLR: 2.0<br>NLR: 0.12<br><br><u>TBI</u><br>Sens: 96%<br>Spec: 50%<br>PLR: 1.9<br>NLR: 0.09	<u>AP</u><br>Sens: 91%<br>Spec: 53%<br>PLR: 2.0<br>NLR: 0.16<br><br><u>TP</u><br>Sens: 94%<br>Spec: 43%<br>PLR: 1.7<br>NLR: 0.10	16% of patients had revascularization before amputation.<br><br>Primary amputation level was below the ankle in 92 patients and above the ankle in 67 patients.
Mehta 1980[53]	United States of America	Prospective observational study<br><br>Follow-up: maximum of 30 months	N = 44 DFU<br><br>Mean age: 59 years<br>Gender: 100% male	45/60 total amputations healed (77% in diabetics)	Definition of healing not specifically mentioned	AP > 60 mmHg	<u>AP</u><br>Sens: 12%<br>Spec: 90%<br>PLR: 1.2<br>NLR: 0.98		Diagnostic performance is calculated based on data from figure.<br><br>Only subgroup analysis (DM) for healed amputation was displayed.
Vitti 1994[54]	United States	Retrospective cohort<br><br>Follow-up time unclear	N = 136 men<br><br>Mean age: 64.8 years old<br>Male: 100%<br><br>DM: n = 110 (81%)	Open wounds after surgery<br><br>Primarily closed wounds after surgery	Non-healing was defined as incisional breakdown requiring reamputation or failure of secondary wound healing in open amputations.	TP ≥ 38 mmHg in non-revascularized diabetics	<u>TP</u><br>Sens: 100%<br>Spec: 56.8%<br>PLR: 2.3<br>NLR: -		Cut-off value was not pre-specified but based on outcomes in population.<br><br>Unclear in how many cases toe used for measurement was amputated. TP only measured pre-operative.
Welch 1985[55]	United Kingdom	Prospective cohort<br><br>Follow-up time unclear	N = 50 minor foot/ transmetatarsal amputations<br><br>Mean age: 61 years<br>Male: 58%<br>Diabetic: 68%, n = 34	No wound classification provided	Primary healing: Clean, dry, healed wound on day 14 post-operatively.<br><br>Delayed healing: Requiring continued treatment without	AP ≥ 40 mmHg<br><br>AP ≥ 60 mmHg	<u>AP (40)</u><br>Sens: 88.0%<br>Spec: 11.1%<br>PLR: 1.0<br>NLR: 1.1<br><br><u>SBF (12)</u><br>Sens: 54.5%	<u>AP (60)</u><br>Sens: 80.0%<br>Spec: 22.2%<br>PLR: 1.0<br>NLR: 0.9<br><br><u>SBF (16)</u><br>Sens: 27.3%	PLR could not be calculated for SBF and the chosen cut-off values because there was no non-healing above the chosen cut-off value. Lower cut-off

					necessity for higher amputation level.<br><br>Non-healing: Necessity to perform higher level amputation or revascularization	SBF ≥ 12 ml 100g^{-1} min^{-1}<br><br>SBF ≥ 16 ml 100g^{-1} min^{-1}	Spec: 100%<br>PLR: inf<br>NLR: 0.5	Spec: 100%<br>PLR: inf<br>NLR: 0.7	values could not be selected because necessary data could not reliably be distinguished from the figures.<br><br>Small sample size.<br><br>Prognostic performance was calculated*.
Wyss 1988[25]	United States	Prospective cohort<br><br>Follow-up time unclear	N = 89 diabetics<br><br>Mean age: 64 years<br>Male: not provided<br><br>112 amputations → 26 foot amputations. Other amputations were major amputations.	Amputation wounds	Healing defined as wound closure with or without additional debridement irrespective of time necessary to heal.<br><br>Failure defined as necessity of major surgical revision.	$TcPO_2$ ≥ 31 mmHg	<u>$TcPO_2$</u><br>Sens: 84.2%<br>Spec: 85.7%<br>PLR: 5.9<br>NLR: 0.2		Large population but only small sample fits criteria of this review (26 foot amputations which are either forefoot or Syme amputations)<br><br>Definition of major surgical revision unclear.<br><br>Many major confounders not assessed or reported.<br><br>Prognostic performance was calculated*.
Zhang 2019[56]	China	Prospective cohort<br><br>Mean follow-up 38 months.	N = 97 DFU<br><br>Mean age: 67 years old<br>Male: 62%<br>Mean DM-duration: 13 years	No specific grading system regarding the DFU<br><br><u>Overall</u><br>65% healing after median of 8	Wound healing after transmetatarsal amputation defined as complete re-epithelialization of the wound	ABI > 0.4<br><br>ABI ≥ 0.7<br><br>ABI > 0.9	<u>ABI (0.4)</u><br>Sens: 98.4%<br>Spec: 23.6%<br>PLR: 1.3<br>NLR: 0.07	<u>ABI (0.7)</u><br>Sens: 81.0%<br>Spec: 82.4%<br>PLR: 4.6<br>NLR: 0.2	In the ABI > 0.9 group the PLR could not be calculated due to no patients with an ABI > 0.9 experiencing

		months (range 1 – 24 months)		ABI (0.9)<br>Sens: 12.7%<br>Spec: 100%<br>PLR: inf<br>NLR: 0.87	failure to heal of the wound.<br><br>Prognostic performance was calculated*.

Abbreviations: ABI = ankle branchial index, AP = ankle pressure, DFU = diabetic foot ulcer, DM = diabetes mellitus, Inf = infinite, PLR = positive likelihood ratio, NLR = negative likelihood ratio, PVR = pulse volume recordings, SABP = systolic ankle pressure, SBF = skin blood flow, SDBP = systolic digital blood pressure, sens = sensitivity, spec = specificity, SPFF = skin perfusion pressure on the feet, TBI = toe brachial index, TcPO2 = transcutaneous oxygen pressure, TP = toe pressure

*** =** Predictive capabilities were calculated based on numbers provided in the article. If no clear cut-off values were mentioned, we chose cut-off values based on
1) how clearly we could distinguish the necessary data from the figures or text and
2) the cut-off values commonly used in other articles.
